# Safety, Efficacy, and Patient-Reported Outcomes of the PureWick™ System Versus Comparator for Nocturnal Urinary Incontinence in the Home Setting: Results of a Randomized Trial

**DOI:** 10.3390/jcm14248699

**Published:** 2025-12-09

**Authors:** Jorge Calle Medina, Mona Fakih, Satkirin Khalsa, Danielle Redmond, Michael J. Kennelly

**Affiliations:** 1 Finlay Medical Research, 6803 Lake Worth Rd, Suite 300, Greenacres, FL 33467, USA; jcalle@finlaymr.com; 2 Women Health Associates, Revive Research Institute, Dearborn Heights, MI 48127, USA; mona.y.fakih@gmail.com; 3 Albuquerque Clinical Trials, 711 Encino Pl NE, Albuquerque, NM 87102, USA; skhalsa@abqct.com; 4 Becton, Dickinson and Company, 8195 Industrial Blvd, Covington, GA 30014, USA; 5 Carolinas Medical Center, Wake Forest School of Medicine, Charlotte, NC 28207, USA; michael.kennelly@wfusm.edu

**Keywords:** female urinary incontinence, nocturnal enuresis, female external catheter, non-invasive catheter, PureWick System, urine management device, home-based care

## Abstract

**Background/Objectives**: Urinary incontinence (UI) is highly prevalent among women and substantially impacts quality of life (QoL). However, there remains an unmet need for non-invasive, easy-to-use urine output management options. This study aimed to evaluate the safety, efficacy, and patient-reported outcomes of a suction-assisted female external wicking catheter system (the PureWick System) compared to a well-established comparator—the Hollister Female Urinary Pouch—for nocturnal UI in the home setting. **Methods**: In total, 165 women (mean age 71.1 years) requiring urine output management at night were randomized 2:1 to use either PureWick (*n* = 107) or Hollister (*n* = 58) each night for four weeks. Primary endpoints were urine capture rate and skin irritation (Draize Scale). Secondary endpoints assessed tolerability, comfort, and ease of use. Exploratory endpoints evaluated QoL (Nocturia-QoL questionnaire) and sleep quality (PROMIS Sleep Disturbance). The trial was designed and powered for non-inferiority; superiority analyses were exploratory [ClinicalTrials.gov: NCT06666426]. **Results**: PureWick met the non-inferiority criteria for both primary endpoints (*p* < 0.0001). Average urine capture rates were similar between PureWick and Hollister (mean: 90.9% vs. 87.9%; median: 95.4% vs. 97.3%), and mean Draize Scale scores were low for both devices (0.02 vs. 0.16). Participants tolerated PureWick for the same duration as Hollister and rated it more comfortable and easier to use. Both devices improved QoL and sleep quality. **Conclusions**: PureWick offers a safe, effective, and patient-friendly option for independently managing nocturnal UI in the home setting. Compared with an established alternative, it achieved non-inferiority for primary efficacy and safety endpoints while offering greater comfort and ease of use.

## 1. Introduction

Urinary incontinence (UI) is the most common pelvic floor disorder among women, with most women in the United States (US) experiencing UI at some point during their lifetime [1,2]. Approximately 62% of adult women in the US reported experiencing UI [2], with the highest rates observed among women aged 65 years or older [1,3,4,5]. However, UI is associated with considerable social stigma [6], which may contribute to under-reporting and under-treatment [7,8]. Women are also less likely to seek professional help for UI compared with men [9].

Underlying causes of UI vary according to its subtype. Stress UI is primarily associated with dysfunction of the internal urethral sphincter, weakness of the pelvic floor musculature, relaxation of supportive fascia and ligaments, and increased urethral mobility. In contrast, urgency UI results from abnormal bladder function, most commonly due to detrusor overactivity, impaired detrusor compliance, and heightened bladder responsiveness [1].

UI symptoms have a substantial impact on quality of life (QoL) and are associated with personal and societal burden [1]. Women with UI experience worse QoL than women without UI symptoms, citing discomfort and sleep disturbance as key drivers [10,11]. UI is also associated with significantly increased risk of falls [12,13], further impacting QoL. Furthermore, women aged 65 years or older are also more likely to sustain a fall-related injury [14].

UI and related loss of independence are known risk factors for nursing home admissions [15,16]. While at-home care can provide greater independence and comfort, there are currently several barriers, and managing UI at home can place a substantial burden and cost on individuals and their caregivers [17].

Current management approaches for UI include non-surgical options (such as pelvic floor muscle strengthening, lifestyle modifications, medications, and indwelling catheters) and surgical options to support the urethra or increase bladder capacity [1,18]. There is currently a paucity of external urine management options for women with UI, likely given the challenge of designing a device that effectively contains urine while avoiding damage to the pubic skin or vaginal mucosa [19]. Absorbent products such as pads and liners are commonly used, and while they can be effective, they are associated with stigma and complications such as incontinence-associated dermatitis and require frequent changes, which can lead to sleep disruptions, reduced comfort, and increased risk of falls [20]. Therefore, there is currently an unmet need for non-invasive, easy-to-use urine output management options that women can use independently in the home setting.

This randomized study aimed to evaluate the safety, efficacy, and patient-reported experience of the PureWick System compared to a well-established comparator—the Hollister Female Urinary Pouch (FUP)—in women requiring urine output management overnight in the home setting. With its novel home-use randomized design and incorporation of patient-reported outcomes, this study addresses gaps in existing literature primarily focused on acute care settings.

## 2. Materials and Methods

### 2.1. Study Design

This was a post-market, prospective, multi-site, open-label, randomized, parallel-arm study. Eligible participants were adult females (≥18 years) who required overnight urine output management at home and currently used absorbent pads or equivalent products at night. Participants were required to provide informed consent and agree to comply with all study procedures.

The exclusion criteria were as follows:

- Frequent episodes of bowel incontinence without a fecal management system in place;
- Moderate to heavy menstruation and cannot use a tampon or menstrual cup;
- Urinary tract, vaginal or other chronic infections or active genital herpes;
- Urinary retention;
- Agitated, combative, and/or uncooperative and may remove the external catheter or pouch;
- Any wound, open lesion or irritation on the genitalia, perineum, or sacrum;
- Any pre-existing neurological, psychiatric, or other condition that would confound QoL assessment or would make it difficult to self-report on QoL questionnaires in the opinion of the investigator;
- Known to be pregnant at time of enrollment (for women of childbearing age);
- Any other condition that, in the opinion of the investigator, would preclude them from participating in the study.

Participants were recruited from sites across the United States (US). After informed consent was obtained and eligibility criteria were verified to be met, participants were randomized 2:1 to use the PureWick System or Hollister FUP by the site using a computer-generated schedule created by an independent third party. Randomization was implemented via Interactive Response Technology (IRT) and stratified by age group (18–64, 65–79, ≥80) and investigational site to ensure balance across key demographic factors.

The study was conducted in accordance with the FDA’s draft guidance on Diversity Action Plans (DAPs) (June 2024) [21], developed in good faith to promote representative enrollment across age, race, and ethnicity. Although not required under the Food and Drug Omnibus Reform Act (FDORA), a DAP was designed to enhance the generalizability of findings to the Medicare population and inform recruitment strategies accordingly [22].

Enrolled participants were trained by nurses at home using standardized training videos available from the manufacturer for the applicable product at baseline and had access to the instructions for use to review throughout the study. Participants were instructed to self-place the assigned device overnight for a period of four weeks (28 days); assistance by a non-professional caregiver (e.g., family member) was allowed. The presence of caregiver support for activities of daily living did not exclude participation in the study. Nurses visited participants’ homes to record device use (including whether the device was placed independently or with assistance), collect urine weight measurements, and assess for skin irritation at baseline and during week 1 (nights 1–5) and week 4 (nights 24–28) of treatment using the Draize Scale (see Table S3 in Supplementary Materials for scoring criteria). On days when nurses did not visit the participants’ homes (weeks 2–3; nights 6–23), a telephone visit was conducted to document whether the assigned treatment had been used during the prior night’s sleep and to report any adverse events.

### 2.2. Treatment

#### 2.2.1. Suction-Assisted Female External Wicking Catheter System

The PureWick^TM^ System (Becton Dickinson, Franklin Lakes, NJ, USA) is a non-invasive, external device. The system includes a Female External Catheter (FEC), a non-sterile, single-use device made of a flexible contoured external catheter (a “wick”). The FEC sits outside the body, where it is held in place by the anatomy. The wick is connected to a free-standing collection canister via tubing. The collection system uses low-pressure suction to pull voided urine through the wick and into the connected collection canister. Peri-care is the recommended patient preparation for FEC application.

**Figure 1:**
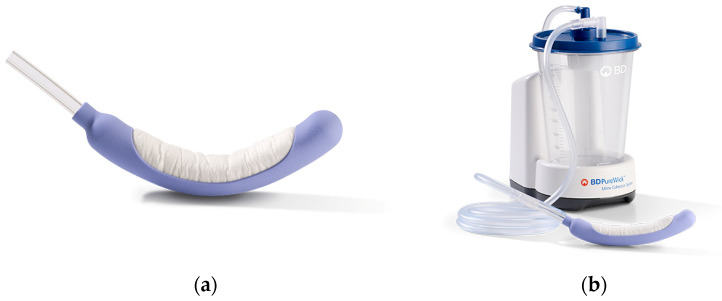
(**a**) PureWick Female External Catheter; (**b**) The PureWick System.

In this study the PureWick System included the PureWick Flex FEC (model: PWFXH30) and PureWick Urine Collection System (model: PW100) (Figure 1).

#### 2.2.2. Female Urinary Pouch

**Figure 2:**
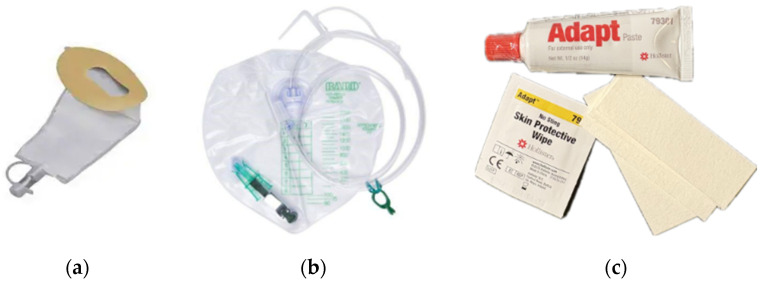
(**a**) Hollister Female Urinary Pouch; (**b**) Bedside Drainage Bag; (**c**) Adapt^TM^ Skin Barrier Paste, skin protectant, and tape strips.

The Hollister FUP (Hollister Inc., 2000 Hollister Dr., Libertyville, IL, USA) is a single-use, cut-to-fit, self-adhering pouch made with hydrocolloid skin adhesive and polymer/copolymer plastics. The pouch is designed to be emptied and to be attached to a drainage bag (Figure 2a,b).

In this study the Hollister FUP with attached SoftFlex^TM^ skin barrier was used. Patient preparation included peri-care, trimming pubic hair, optional application of skin protectant, cutting the device opening to fit, and application of Adapt^TM^ Skin Barrier Paste to help with device fit (Figure 2c). All ancillary products used in the training video were provided to participants (including trimmers/hair clippers, skin protectant, Adapt paste, and microporous adhesive strips for additional adhesive).

#### 2.2.3. Rationale for Comparator Selection

The Hollister FUP was selected as the comparator in this study due to its established clinical use, safety profile, and relevance to the study objectives. It is a well-known, commercially available external urinary management device that is currently reimbursed by Medicare, making it a practical and accessible option in real-world clinical settings.

Moreover, the FUP has been evaluated in long-term care settings and demonstrated favorable outcomes in terms of leakage control, patient tolerability, and reduced incidence of bacteriuria compared to indwelling urethral catheters. The incidence of new bacteriuria was significantly lower than that observed with long-term catheter use, and adverse skin reactions were minimal and typically self-limiting. These findings support the FUP’s appropriateness as a benchmark for evaluating alternative urinary management solutions in incontinent female populations [23].

### 2.3. Objectives and Endpoints

The primary objective was to compare the efficacy and safety of the two treatment devices. Efficacy was measured based on the daily urine capture rate calculated using captured and leaked urine weight measurements collected during in-home assessments (captured urine weight/[captured urine weight + leaked urine weight] * 100). Safety was assessed by skin irritation using the Draize Scale [24] score evaluated by nurses during in-person assessments on weeks 1 and 4. The nurses had received standardized training on the protocol and Draize Scale, including multiple case studies to ensure scoring consistency. Inter-observer variability was not formally evaluated. If a nurse encountered scoring-related questions or deemed it necessary, a photograph of the skin irritation site was submitted to the investigator for review. During weeks 2 and 3, participants self-reported on skin irritation during telephone visits. The secondary objectives were to assess tolerability, comfort, and ease of use. Tolerability was measured by the number of days of actual use of each device during the four-week period, recorded through a participant-completed log, phone assessments, and in-home assessments. Comfort and ease of use were measured using subjective evaluations of the devices via participant surveys (5-point Likert scales) collected at the end of treatment.

For the exploratory objectives, changes in self-reported QoL were measured using the validated Nocturia Quality of Life (N-QoL) questionnaire at baseline, after device use on night 14, and at the end of treatment, and sleep quality was measured using the Patient-Reported Outcomes Measurement Information System (PROMIS^TM^) Sleep Disturbance Short Form (SF) 4A at baseline and after device use on nights 7, 14, and 21.

The N-QoL questionnaire consists of 13 items scored from 0 to 4 (0 = lowest QoL, 4 = highest QoL). The raw total score was the sum of the first 12 questions (with question 13, the global QoL item, scored separately). The raw total score was transformed onto a standardized scale of 0–100. Higher scores indicate a higher QoL. The N-QoL questionnaire has been validated and applied in similar populations in prior studies [25,26,27]. The N-QOL questionnaire was originally developed by Pfizer Inc. and is used under license from Cronos Clinical Consulting Services, Inc. All licensing and permission requests should be directed to IQVIA Inc. at COAsolutions@iqvia.com. The PROMIS Sleep Disturbance SF 4A consists of 4 items with participants rating aspects of their sleep over the past 7 days on 5-point Likert scales. The raw total score was transformed into a T-score using the conversion table in the PROMIS sleep scoring manual [28]. The T-score rescales the raw score into a standardized score with a mean of 50 and a standard deviation (SD) of 10. A score of 50 is the average for the US general population with a standard deviation of 10 because calibration testing was performed on a large sample of the general population. For the sleep disturbance scale, a negatively worded concept, T-scores of 60 are one SD worse than average. By comparison, T-scores of 40 are one SD better than average [28]. T-scores for the PROMIS Sleep Disturbance SF 4a range from 32 to 73.3, with lower scores indicating less sleep disturbance. The precision and efficiency of the PROMIS sleep disturbance short form have previously been demonstrated [29].

### 2.4. Statistical Analyses

Sample size determination was based on the statistical power required for the co-primary and secondary endpoints, as well as a 2:1 randomization ratio between the PureWick System and Hollister FUP groups. To ensure adequate power across both co-primary endpoints and key secondary endpoints, a total sample size of 150 participants was selected. For the efficacy co-primary endpoint (urine capture rate), a sample size of 150 provided 99% power to demonstrate non-inferiority using a margin of 10% (one-sided alpha = 0.025). For the safety co-primary endpoint (skin irritation), a sample size of 150 provided 99% power to demonstrate non-inferiority using a margin of 1.2 points (one-sided alpha = 0.025). The planned sample size provided 87% power to demonstrate superiority in capture rate, assuming the average capture rate of the PureWick System was 70%, the average capture rate of the Hollister FUP was 55%, the standard deviation of the capture rate was 25%, and the attrition rate was approximately 20%. Refer to Supplementary Materials S1 for further details. Missing diary entries and patient-reported outcome questionnaires were excluded from analysis; no imputation was performed. Sensitivity analyses were not conducted.

This study was powered for non-inferiority for the co-primary endpoints (urine capture rate and skin irritation). Superiority testing was exploratory and only conducted in a hierarchical stepwise manner after non-inferiority was confirmed, as per the gatekeeping procedure.

Hypothesis testing was conducted for the co-primary and secondary endpoints. To control the study-wise Type 1 error rate, the Gatekeeping method was used in combination with both the truncated and conventional Hochberg procedure [30]. The co-primary endpoints were tested using a one-sided alpha of 0.025. If both co-primary endpoints passed hypothesis testing, any unused alpha was carried forward for superiority testing of the capture rate. However, if either co-primary endpoint failed, further testing was not performed. Refer to Supplementary Materials S1 for further details.

The intent-to-treat (ITT) population, comprising all participants who were randomized to a treatment group, was used for analyses of endpoints. The as-treated (AT) population, comprising the ITT population grouped by actual treatment, was used for analyses of safety outcomes. Primary endpoint analyses were based on available data.

Analyses were performed using SAS Version 9.4 or above (SAS Institute, Cary, NC, USA).

### 2.5. Ethics and Consent

The study was reviewed and approved by the Ethics Committee, Advarra Institutional Review Board (IRB). The protocol adhered to the ethical principles of the Declaration of Helsinki for medical research involving human subjects. Investigators did not receive any incentives or benefits for participant recruitment, as documented in the IRB-approved protocol (Advarra #Pro00084784). Participants received a modest stipend for study-related activities beyond routine care requirements. The stipend was paid in milestones based on fair market value and was approved by the IRB as part of the protocol. The study was registered at ClinicalTrials.gov (NCT06666426).

## 3. Results

### 3.1. Study Population and Protocol Deviations

**Figure 3:**
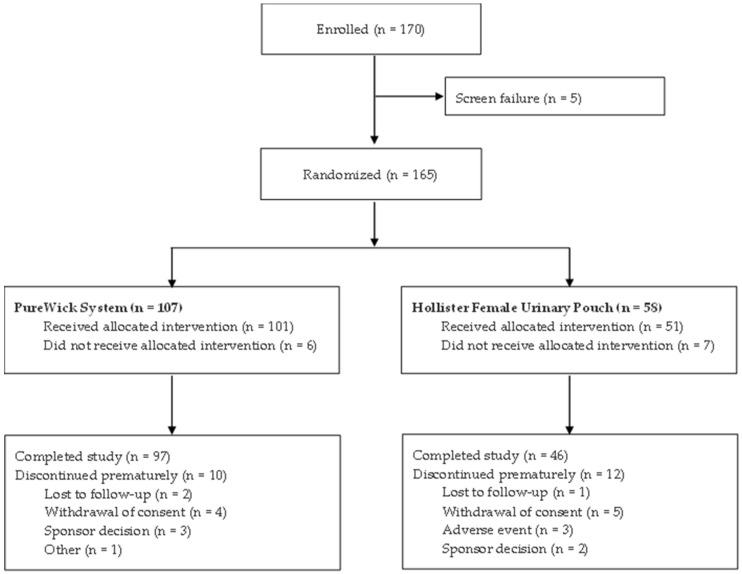
Participant flow diagram.

Between October 2024 and June 2025, 170 participants were enrolled from 13 sites across the US. Of these, 165 were randomized to either the PureWick System (*n* = 107) or Hollister FUP (*n* = 58) treatment group (Figure 3). A total of 143 (86.7%) participants completed the study, and 22 (13.3%) discontinued prematurely. Of note, no participants discontinued using the PureWick System due to adverse events, in comparison to 3 participants in the Hollister FUP group. Of those, 2 participants experienced erythema (one causal relationship to study device/procedures, one probable relationship to study device/procedure) and one participant experienced a urinary tract infection, which was not related to the study device or procedures.

Demographic and baseline characteristics were well balanced between groups (Table 1). All participants were female, and the mean age was 71.3 years. A primary diagnosis of UI was most prevalent (65.5%), followed by nocturia (20.6%). The primary diagnosis was defined as the (self-reported) primary reason the participant needed urinary management overnight.

To enhance the generalizability of the study population to the Medicare demographic, enrollment of participants aged 18 to 64 years was capped at 12%. As a result, 89.1% (147/165) of enrolled participants were aged 65 years or older, while only 10.9% (18/165) were within the 18 to 64 age group (mean age 58.1 years). Within the PureWick group, 96 of 107 participants (89.7%) were aged 65 years or older, and 11 participants (10.3%) were aged 18 to 64 years. In the Hollister FUP group, 51 of 58 participants (87.9%) were aged 65 years or older, and 7 participants (12.1%) were aged 18 to 64 years.

Four participants were withdrawn following a sponsor decision made in consultation with the site investigator. One participant in the PureWick group underwent a planned elective surgery after enrollment and required unanticipated prolonged hospitalization with an indwelling urinary catheter, which precluded completion of study visits. Additionally, two participants in the PureWick group and two in the Hollister group were enrolled at a site experiencing nursing staff turnover; a qualified nurse was not hired in time to conduct the study visits. These withdrawals were administrative and unrelated to device safety or performance.

A total of 115 protocol deviations were reported by 38 participants during the study, none of which were classified as major or related to device safety (Table 2). The proportion of participants with at least one protocol deviation was comparable between groups (23.4% for PureWick vs. 22.4% for Hollister). Most deviations were attributable to a missed clinical assessment, followed by clinical assessments being performed out of the predefined time window and missed visits. All protocol deviations classified as ‘Other’ were minor and generally involved patient-related issues such as incorrect device use, device disposal, or insufficient sample collection.

### 3.2. Urine Capture Rate

The primary efficacy endpoint was daily urine capture rate calculated using captured and leaked urine weight measurements. The average urine capture rate was similar between the PureWick System and the Hollister FUP (mean: 90.9% vs. 87.9%; median: 95.4% vs. 97.3%) (Table 3). The comparison between the PureWick System and Hollister FUP met the criterion for non-inferiority (*p* < 0.0001) (Table 4), based on the prespecified margin of 10%. While the results demonstrated non-inferiority of the PureWick System compared to the Hollister FUP, exploratory statistical superiority was not demonstrated (*p* = 0.143).

### 3.3. Skin Irritation (Draize Scale)

The primary safety endpoint was skin irritation measured using the Draize Scale. The incidence of erythema, edema, and bleeding was minimal across both device groups, with scores greater than zero occurring infrequently (Table 5). The average Draize Scale score was similar with the PureWick System and Hollister FUP (0.02 vs. 0.16). The comparison between the PureWick System and Hollister FUP met the criterion for non-inferiority (*p* < 0.0001), based on the prespecified margin of 1.2 points (Table 6).

### 3.4. Tolerability

On average participants used the PureWick System for 26.9 out of 28 days and the Hollister FUP for 25.1 out of 28 days (Table 7). Between groups the mean difference (PureWick compared to Hollister) was 1.8 days. The exploratory superiority test did not demonstrate statistical significance (*p* = 0.0252).

### 3.5. Participant Comfort, Ease of Use and Overall Opinion

Participant ease of use and comfort were measured using 5-point Likert scales at the end of treatment. On average, participants reported higher scores with the PureWick System compared to the Hollister FUP for ease of device placement (4.5 vs. 3.4) and ease of device removal (4.7 vs. 2.4). Between groups the mean difference (PureWick compared to Hollister) was 1.1 (*p* < 0.0001) for ease of device placement and 2.3 (*p* < 0.0001) for ease of device removal (Table 8), demonstrating that participants found the PureWick System easier to place and remove than the Hollister FUP. Based on the multiplicity control rules defined in Supplemental Material S1, such findings can only be interpreted as exploratory.

On average, participants also reported higher scores with the PureWick System compared to the Hollister FUP for comfort of device placement (3.9 vs. 2.9), comfort during sleep (3.8 vs. 3.0), and comfort of device removal (4.3 vs. 2.2). Between groups the mean difference (PureWick compared to Hollister) was 1.0 (*p* < 0.0001) for comfort of device placement, 0.7 (*p* = 0.0003) for comfort during sleep, and 2.1 (*p* < 0.0001) for comfort of device removal (Table 8), demonstrating that participants found the PureWick System more comfortable to place, use during sleep, and remove than the Hollister FUP. Based on the multiplicity control rules defined in Supplemental Material S1, such findings can only be interpreted as exploratory.

Participant overall opinion on the urine collection devices was also measured using a short questionnaire at the end of study treatment. Overall, the majority of participants (69.4%; 68/98) stated that they would like to use the PureWick System beyond the study, compared to less than half of participants for the Hollister FUP (40.8%; 20/49). Similarly, the majority of participants (89.9%; 88/98) stated that they would be Very Likely or Likely to recommend the PureWick System to a loved one, compared to about half of participants for the Hollister FUP (53.1%; 26/49).

### 3.6. Exploratory Analysis of Quality of Life (N-QoL) and Sleep Quality (PROMIS Sleep Disturbance)

Self-reported changes in QoL and sleep quality were measured as exploratory outcomes using the N-QoL questionnaire and PROMIS Sleep Disturbance Short Form, respectively. For the N-QoL, the mean change from baseline to the end of study treatment increased for both groups, indicating an improvement in QoL: 29.9 (*n* = 97) PureWick and 28.6 (*n* = 48) Hollister. For the PROMIS Sleep Disturbance scale, the mean total T-score change from baseline to the end of treatment decreased for both groups, indicating improvement in sleep quality: −8.61 (*n* = 98) PureWick and −6.8 (*n* = 48) Hollister.

### 3.7. Safety Outcomes

For the PureWick System group, 9 adverse events by 7 (6.9%; 7/101) participants were reported, with the majority (7/9 events) “not related” to the device or procedure. Two events had a “possible” relationship to the device or procedure (vulvovaginal injury and diaper dermatitis); however, both were mild in severity. The vulvovaginal injury was treated with topical zinc oxide (Desitin^®^) before and after device use, and the condition resolved without sequelae. Two serious adverse events by 2 participants (2.0%) were reported; however, both were “not related” to the device or procedure. For the Hollister FUP group, 8 adverse events by 7 (13.7%; 7/51) participants were reported, with 5 events reported as being related to the study device or procedure. Of the device/procedure-related events, 4 were due to erythema, and the other was due to a visible spot of red blood. No deaths, unanticipated adverse device effects, or serious health threats were reported in this study (Table 9).

In total, 2 device deficiencies from 2 participants were reported in the study, both in the PureWick System group (detachment of parts during sleep and device dropped by participant before placement); however, these were not associated with an adverse event, could not have led to a serious adverse device effect if suitable action had not been taken, and did not meet the definition of a serious health threat.

## 4. Discussion

The present study assessed the efficacy, safety, and patient-reported outcomes of participants requiring urine output management overnight in the home setting. In this prospective, open-label, randomized, parallel-arm study, participants were randomized 2:1 to use either the PureWick System or Hollister FUP overnight and followed for four weeks. Participants were instructed to report any discomfort or voiding issues occurring within 24 h of treatment completion. The PureWick System met the non-inferiority criteria for both primary efficacy (urine capture rate) and safety (Draize Scale for skin irritation) endpoints.

Importantly, participants were able to place the devices independently in the home setting during the study, in the absence of a professional caregiver. The data show that 96.8% (947/978) of placements were completed by the participant in the PureWick group and 91.3% (420/460) for the Hollister FUP group. Participants also found the PureWick System easier to use and more comfortable than the Hollister FUP. This supports the use of the PureWick System in the home setting, with the potential to reduce the burden on caregivers and increase independence and comfort for individuals. While exploratory findings suggest potential improvements in QoL and sleep quality, these outcomes were not adjusted for multiplicity and should be interpreted cautiously. In a recent conjoint analysis, Welk et al. (2022) showed that the frequency of awakenings at night and feeling rested in the morning were aspects that are significantly important to women with nocturnal enuresis [11].

The findings of this present study support data from previous clinical studies [20,31] while increasing generalizability to the US Medicare population. Participants were enrolled from 13 sites across the US, covering a range of geographic locations and suburban, urban, and rural populations. Demographic and baseline characteristics were diverse in ethnicity and race and were well balanced between the treatment groups. Furthermore, the study population mirrors that of the Medicare population, with 89.1% aged 65 years or older, supporting generalizability of the findings.

Several limitations should be considered when interpreting these findings. This study was a decentralized study conducted in the home setting. The study relied on participants and home nurses to follow protocol-defined procedures. Some of these procedures exceed what would typically be required for device use in the real-world setting, increasing the risk of protocol deviations and anomalies (see Supplementary Materials S2). Indeed, in this study 38 participants (23.0%) had at least one protocol deviation. While these deviations may introduce variability, they reflect the real-world challenges of conducting clinical research outside traditional care environments. Furthermore, at one site, incomplete data documented by the nurse led to the exclusion of four consecutive participants from the PureWick group from the primary endpoint analysis. The missing weight measurements prevented calculation of urine capture rate. Consequently, additional protocol training was completed. For the urine capture rate, the timing of bed pad weight measurements can vary due to scheduling constraints; however, bench testing showed that weight loss due to evaporation was minimal. The reasons for low urine capture were not analyzed in the present study. Potential contributing factors include body habitus, urinary flow rate, voided volume, sleeping position, and patient movement during sleep. Device attachment and patient education may also influence performance; however, these were standardized across both groups. Future studies should incorporate these variables to better understand capture variability.

Furthermore, when interpreting patient-reported outcomes, potential confounders should be considered. Specifically, the unblinded design, variability in caregiver assistance, and differences in training intensity across sites may have influenced subjective measures such as ease of use and comfort. These limitations highlight real-world variability and the potential for bias and reinforce the importance of robust data collection procedures in home-based clinical research. Future research is warranted to examine long-term outcomes, caregiver workload, catheter-associated urinary tract infection rates, and cost and resource utilization to enhance clinical relevance.

Despite its limitations, this study made significant strides toward advancing the understanding of the PureWick System in the community setting. Approximately 70% of the participants reported an intention to continue to use the PureWick System beyond the study, echoing the high patient and nurse satisfaction reported in a recent meta-analysis of predominantly hospitalized patients [32].

These findings contribute to the growing body of evidence supporting the shift in healthcare delivery into patients’ homes. While the device itself has been previously evaluated, the present study contributes unique evidence on usability and patient experience in the home setting. As healthcare systems increasingly prioritize patient autonomy, cost-effectiveness, and decentralized care models—non-invasive, easy-to-use solutions like the PureWick System may play a critical role in enabling individuals to manage chronic conditions independently. Future research should explore the integration of such technologies into broader home care strategies, particularly for aging populations and those with limited access to institutional care.

## 5. Conclusions

The results of this randomized study show that the PureWick System is a safe, effective, and patient-friendly option for independently managing nocturnal UI in the home setting. Compared with the Hollister FUP, an established alternative, it achieved non-inferiority for the primary efficacy and safety endpoints while offering greater comfort and ease of use.

## Figures and Tables

**Table 1:** Demographics and baseline characteristics.

	PureWick (*n* = 107)	Hollister (*n* = 58)	Total (*n* = 165)
Age (years)			
*n*	107	58	165
Mean (SD)	71.1 (7.3)	71.7 (8.4)	71.3 (7.7)
Median	71.0	73.0	71.0
Min–max	51–92	45–88	45–92
Sex			
Female	107 (100%)	58 (100%)	165 (100%)
Ethnicity			
Hispanic or Latino	46 (43.0%)	24 (41.4%)	70 (42.4%)
Not Hispanic of Latino	57 (53.3%)	31 (53.4%)	88 (53.3%)
Not reported	1 (0.9%)	3 (5.2%)	4 (2.4%)
Unknown	3 (2.8%)	0 (0.0%)	3 (1.8%)
Race			
Black or African American	40 (37.4%)	23 (39.7%)	63 (38.2%)
White	65 (60.7%)	32 (55.2%)	97 (58.8%)
Unknown	2 (1.9%)	3 (5.2%)	5 (3.0%)
Primary diagnosis ^1^			
Urinary incontinence	71 (66.4%)	37 (63.8%)	108 (65.5%)
Nocturia	23 (21.5%)	11 (19.0%)	34 (20.6%)
Reduced mobility	5 (4.7%)	2 (3.4%)	7 (4.2%)
Falls risk/history of falling	1 (0.9%)	1 (1.7%)	2 (1.2%)
Other	1 (0.9%)	0 (0.0%)	1 (0.6%)
Missing	6 (5.6%)	7 (12.1%)	13 (7.9%)
Weight (kg)			
*n*	101	51	152
Mean (SD)	84.7 (20.5)	77.49 (17.4)	82.31 (19.8)
Median	79.0	77.0	78.0
Min–max	48.2–176	49.8–123.3	48.2–176
Height (cm)			
*n*	101	51	152
Mean (SD)	161.2 (9.3)	159.4 (9.3)	160.6 (9.3)
Median	161.5	160.0	160.8
Min–max	121.9–190	137–186	121.9–190
BMI (kg/m^2^)			
*n*	101	51	152
Mean (SD)	32.7 (7.6)	30.6 (6.5)	32.0 (7.3)
Median	31.10	30.40	30.95
Min–max	15.8–66.6	18.5–46.7	15.8–66.6

BMI, body mass index; SD, standard deviation. ^1^ ‘Primary diagnosis’ refers to the self-reported reason participants required overnight urine management. These categories were chosen and mapped to existing ICD-10 codes to provide context for potential reimbursement and future research considerations.

**Table 2:** Protocol deviations.

	PureWick (*n* = 107)	Hollister (*n* = 58)	Total (*n* = 165)
Total number of protocol deviations	74	41	115
Number of participants with at least one protocol deviation	25 (23.4%)	13 (22.4%)	38 (23.0%)
Participant missed follow up visit	6 (5.6%)	1 (1.7%)	7 (4.2%)
Clinical assessment not performed	18 (16.8%)	11 (19.0%)	29 (17.6%)
Clinical assessment out of window	6 (5.6%)	2 (3.4%)	8 (4.8%)
Other	4 (3.7%)	2 (3.4%)	6 (3.6%)
Total number of major protocol deviations	0	0	0
Number of participants with at least one major protocol deviation	0 (0.0%)	0 (0.0%)	0 (0.0%)

**Table 3:** Capture rate summary.

Average of Nightly Capture Rate (%) ^1^	PureWick (*n* = 107)	Hollister (*n* = 58)
*n*	99	48
Mean (SD)	90.9 (13.2)	87.9 (20.8)
Median	95.4	97.3
Min–max	22.5–100	5.9–99.9

^1^ Analysis performed using evaluable voids. SD, standard deviation.

**Table 4:** Non-inferiority test of capture rate.

	Mean Difference	SD	One-Sided 97.5% CI Lower Bound	*p* Value
Difference in average of nightly capture rate (%), PureWick compared with Hollister	3.0	16.1	−2.6	<0.0001

CI, confidence interval; SD, standard deviation. Non-inferiority was achieved if the CI lower bound was greater than the non-inferiority margin of −10%.

**Table 5:** Draize Scale score for skin irritation.

Category	Score	PureWick (*n* = 1065)	Hollister (*n* = 502)
Erythema	0	1054 (99.0%)	482 (96.0%)
	1	6 (0.6%)	17 (3.4%)
	2	5 (0.5%)	2 (0.4%)
	3	0 (0.0%)	0 (0.0%)
	4	0 (0.0%)	1 (0.2%)
Edema	0	1065 (100.0%)	501 (99.8%)
	1	0 (0.0%)	1 (0.2%)
	2	0 (0.0%)	0 (0.0%)
	3	0 (0.0%)	0 (0.0%)
	4	0 (0.0%)	0 (0.0%)
Bleeding	0	1060 (99.5%)	500 (99.6%)
	1	5 (0.5%)	1 (0.2%)
	2	0 (0.0%)	1 (0.2%)
	3	0 (0.0%)	0 (0.0%)
	4	0 (0.0%)	0 (0.0%)

**Table 6:** Non-inferiority test of Draize Scale score.

	Mean Difference	SD	One-Sided 97.5% CI Upper Bound	*p* Value
Difference in average of daily Draize Scale total score, PureWick compared with Hollister	−0.14	0.39	−0.01	<0.0001

CI, confidence interval; SD, standard deviation. Non-inferiority was achieved if the CI upper bound was less than the non-inferiority margin of 1.2.

**Table 7:** Tolerability—number of days of actual use.

Number of Days of Actual Use ^1^	PureWick (*n* = 107)	Hollister (*n* = 58)
*n*	101	51
Mean (SD)	26.9 (3.9)	25.1 (7.3)
Median	28.0	28.0
Min–max	1–28	1–28

^1^ Out of a maximum of 28 days. SD, standard deviation.

**Table 8:** Test of participant ease of use and comfort questionnaire.

	Mean Difference	SD	One-Sided 97.5% CI Lower Bound	*p* Value
Ease of use score				
Difference in ease of device placement	1.1	1.0	0.7	<0.0001
Difference in ease of device removal	2.3	1.0	2.0	<0.0001
Comfort of use score				
Difference in comfort of device placement	1.0	1.2	0.5	<0.0001
Difference in comfort of device during sleep	0.7	1.2	0.3	0.0003
Difference in comfort of device removal	2.1	1.1	1.7	<0.0001

CI, confidence interval; SD, standard deviation.

**Table 9:** Summary of adverse events.

	PureWick (*n* = 101) ^1^		Hollister (*n* = 51) ^1^	
	By Event	By Participant	By Event	By Participant
Any adverse events	9	7 (6.9%)	8	7 (13.7%)
Severity				
Mild	5	4 (4.0%)	4	3 (5.9%)
Moderate	3	2 (2.0%)	2	2 (3.9%)
Severe	1	1 (1.0%)	2	2 (3.9%)
Relationship to study device				
Causal	0	0 (0.0%)	2	2 (3.9%)
Probable	0	0 (0.0%)	2	2 (3.9%)
Possible	2	2 (2.0%)	1	1 (2.0%)
Not related	7	5 (5.0%)	3	2 (3.9%)
Relationship to procedure				
Causal	0	0 (0.0%)	2	2 (3.9%)
Probable	0	0 (0.0%)	1	1 (2.0%)
Possible	2	2 (2.0%)	2	2 (3.9%)
Not related	7	5 (5.0%)	3	2 (3.9%)
Serious adverse events	2	2 (2.0%)	1	1 (2.0%)
Relationship to study device				
Not related	2	2 (2.0%)	1	1 (2.0%)
Relationship to procedure				
Not related	2	2 (2.0%)	1	1 (2.0%)
Death	0	0 (0.0%)	0	0 (0.0%)
Unanticipated adverse device effect	0	0 (0.0%)	0	0 (0.0%)
Serious health threat	0	0 (0.0%)	0	0 (0.0%)

^1^ AT population: participants were counted in the highest severity or most related category.

## Data Availability

The data that supports the findings of this study are available from the sponsor upon reasonable request.

## Supplementary Materials

The following supporting information can be downloaded at: https://www.mdpi.com/article/10.3390/jcm14248699/s1, S1: Statistical Considerations; S2: Measurement Anomalies; S3: Draize Scoring Table.

## Abbreviations

The following abbreviations are used in this manuscript:

AT	As treated
CI	Confidence interval
DAP(s)	Diversity Action Plan(s)
FEC	Female external catheter
FUP	Female urinary pouch
IRB	Institutional Review Board
IRT	Interactive Response Technology
ITT	Intent-to-treat
N-QoL	Nocturia Quality of Life
PROMIS	Patient-Reported Outcomes Measurement Information System
QoL	Quality of life
SD	Standard deviation
SF	Short form
UI	Urinary incontinence
US	United States
