# Supplementary material for: Safety, Efficacy, and Patient-Reported Outcomes of the PureWick™ System Versus Comparator for Nocturnal Urinary Incontinence in the Home Setting: Results of a Randomized Trial
Source: J Clin Med. 2025 Dec 9;14(24):8699. doi: 10.3390/jcm14248699 (PMC12734071; doi:10.3390/jcm14248699)
Supplement: Supplementary file 1 [file jcm-14-08699-s001.zip › S1_Statistical Considerations.pdf]

	**Title**: Statistical Analysis Plan Tables, Figures, and Listings	Page No. **1 of 82**
	**Study Title:** A Randomized, Controlled Trial on the Safety, Efficacy, and Patient Reported Experience Comparing PureWick™ System with an Established Comparator Overnight in the Home Setting (PUREST)<br>**CIP/CPSP Version:** 01	Version No: **1.0**

Template GFM-10082B

Tables/Listing/Figures Shell for Final Report

**PREPARED BY:**		
Shuangshuang Fu	17-Jul-2025	SAP Version: 1.0
(Printed Name):	Date: (DD-MMM-YYYY)	

Downloaded on 18 Jul 2025
System Version Number 1.0

Table of Contents

1.0 GENERAL GUIDANCE ON OUTPUT FORMAT 3
1.1 Document Headers and Footnotes 3
1.2 Presentation of Table Numbering and Titles Within Document 3
1.3 Output Format 5
2.0 SHELLS AND SPECIFICATIONS FOR TABLES 5
3.0 SHELLS AND SPECIFICATIONS FOR LISTINGS 65
4.0 Figures 80
5.0 REFERENCES 82
6.0 VERSION HISTORY 82

Downloaded on 18 Jul 2025
System Version Number 1.0

1.0 GENERAL GUIDANCE ON OUTPUT FORMAT

It is suggested that computer-generated output adhere to the following specifications.

1.1 Document Headers and Footnotes

Unless otherwise specified, all computer-generated output should be produced in landscape mode. All output should have the following header at top of page:

BD	Page n of N
CIP: [XXX-XXXX]	
XXX Report	

And the following as footer at bottom of page:

Program name: [xxxxx.sas]	Date [DD-MMM-YYYY: hhmmss]
Data Source: ADSL, ADTL, ADDEV, ADIMFD, etc.	
Cutoff Date: [DD-MMM-YYYY]	

1.2 Presentation of Table Numbering and Titles Within Document

Each output should be identified by a numeral followed by the title. The study population should be identified on the line immediately following the title. (example below.)

Downloaded on 18 Jul 2025
System Version Number 1.0

**Table No.**	**Table Title**
	**Table Title continued (if necessary)**
Study Population	

Other general table formatting:

- Column headings should in initial upper-case characters,
- For numeric variables, include “unit” in column heading when appropriate.
- Only these categories for which there are at least one (1) subject represented in one (1) or more groups should be included.
- An “unknown” or “missing” category should be added to any parameter for which information is not available for one (1) or more subjects.

Downloaded on 18 Jul 2025
System Version Number 1.0

1.3 Output Format

The estimated mean, median and standard deviation (SD) for a set of values should be printed out to one more decimal place than the individual units of measurement. For example, for age, with raw data in whole years, the following precision would be used:

Example Formatting Requirements

n	xx
Mean (SD)	xx.x (xx.x)
Median	xx.x
Min – Max	xx – xx

P -values should be formatted as follows: “0.xxxx”, where xxxx is the value rounded to 4 decimal places. If the p-value is less than 0.0001 then it should be displayed as ‘<0.0001’

Percentage values should be printed with one digit to the right of the decimal point (e.g., 12.8%, 5.4%). Less-than signs “<0.1%” should be printed when values are >0.0% and <0.1% (not 0.0%).

For listings, missing data may be left blank or “N/A,” may be used, whichever is appropriate.

- For tables displaying counts and percent:
- a data column entry of “n (%)” in the template indicates that the “N” in the column header will be used as denominator throughout the table.
- a data column entry of “n/N (%)” in the template indicates that the number of non-missing data values will be used as the denominator, unless a note indicates a different denominator is to be used.

2.0 SHELLS AND SPECIFICATIONS FOR TABLES

Downloaded on 18 Jul 2025
System Version Number 1.0

Table 14.1.1 Subject Disposition
Enrolled

	PureWick	Hollister	Total
Enrolled			n
Screen Failure			n
ITT	n	n	n
Randomized and Not Treated	n(%)	n(%)	n(%)
Randomized and Treated	n(%)	n(%)	n(%)
Completed Study	n(%)	n(%)	n(%)
Discontinued prematurely	n(%)	n(%)	n(%)
Lost to Follow-up	n(%)	n(%)	n(%)
Withdrawal of Consent	n(%)	n(%)	n(%)
Adverse Event	n(%)	n(%)	n(%)
Death	n(%)	n(%)	n(%)

Downloaded on 18 Jul 2025
System Version Number 1.0

Protocol Deviation	n(%)	n(%)	n(%)
Sponsor Decision	n(%)	n(%)	n(%)
Investigator decision	n(%)	n(%)	n(%)
Failure to Meet Continuation Criteria	n(%)	n(%)	n(%)
Other	n(%)	n(%)	n(%)

Programming note:

1. Display reasons of discontinuation reported for at least one subject only.

Table 14.1.2 Screen Failures

Enrolled Subjects

Subjects Enrolled	xxx
Screen Failure	xx (xx.x%)
Inclusion Criteria not Met	xx (xx.x%)
INCL01	xx (xx.x%)
INCL02	xx (xx.x%)
INCL03	xx (xx.x%)

INCL04	xx (xx.x%)
Exclusion Criteria Met	xx (xx.x%)
EXCL01	xx (xx.x%)
EXCL02	xx (xx.x%)
EXCL03	xx (xx.x%)
EXCL04	xx (xx.x%)
EXCL05	xx (xx.x%)
EXCL06	xx (xx.x%)
EXCL07	xx (xx.x%)
EXCL08	xx (xx.x%)
EXCL09	xx (xx.x%)

Table 14.1.3 Analysis Population
Enrolled

	PureWick	Hollister	Total
Enrolled			n

Downloaded on 18 Jul 2025
System Version Number 1.0

Intent-to-Treat (ITT)	n	n	n
As-Treated (AT)	n	n	n
Per-Protocol (PP)	n	n	n

Table 14.1.4 Demographics and Baseline Characteristics
ITT

	PureWick (N=xxx)	Hollister (N=xxx)	Total (N=xxx)
Age (Years)			
N	xxx	xxx	xxx
Mean (SD)	xx.x (xx.xx)	xx.x (xx.xx)	xx.x (xx.xx)
Median	xx.x	xx.x	xx.x
Min – Max	xx - xx	xx - xx	xx - xx
Sex			
Female	n (%)	n (%)	n (%)
Ethnicity			
Hispanic or Latino	n (%)	n (%)	n (%)

Downloaded on 18 Jul 2025
System Version Number 1.0

Not Hispanic or Latino	n (%)	n (%)	n (%)
Not Reported	n (%)	n (%)	n (%)
Unknown	n (%)	n (%)	n (%)
Race			
White	n (%)	n (%)	n (%)
……	n (%)	n (%)	n (%)
Two or More Races	n (%)	n (%)	n (%)
Primary Diagnosis			
Urinary Incontinence	n (%)	n (%)	n (%)
…	n (%)	n (%)	n (%)
Other	n (%)	n (%)	n (%)
Medical History			
Yes	n (%)	n (%)	n (%)
Weight (kg)			
N	xxx	xxx	xxx

Downloaded on 18 Jul 2025
System Version Number 1.0

Mean (SD)	xx.x (xx.xx)	xx.x (xx.xx)	xx.x (xx.xx)
Median	xx.x	xx.x	xx.x
Min – Max	xx - xx	xx - xx	xx - xx
Height (cm)			
N	xxx	xxx	xxx
Mean (SD)	xx.x (xx.xx)	xx.x (xx.xx)	xx.x (xx.xx)
Median	xx.x	xx.x	xx.x
Min – Max	xx - xx	xx - xx	xx - xx
BMI (kg/m^2)			
N	xxx	xxx	xxx
Mean (SD)	xx.x (xx.xx)	xx.x (xx.xx)	xx.x (xx.xx)
Median	xx.x	xx.x	xx.x
Min – Max	xx - xx	xx - xx	xx - xx
Any Skin Irritation			
Yes	n (%)	n (%)	n (%)

Downloaded on 18 Jul 2025
System Version Number 1.0

No	n (%)	n (%)	n (%)

Table 14.1.5 Protocol Deviations

ITT

	PureWick (N=xxx)	Hollister (N=xxx)	Total (N=xxx)
Total Number of Protocol Deviations	n	n	n
Number of Subjects with at Least One Protocol Deviation	n(%)	n(%)	n(%)
Subject Missed Follow Up Visit	n(%)	n(%)	n(%)
Clinical Assessment Not Done	n(%)	n(%)	n(%)
Randomization Error	n(%)	n(%)	n(%)
Informed Consent	n(%)	n(%)	n(%)
Safety Reporting	n(%)	n(%)	n(%)
Clinical Assessment Out of Window	n(%)	n(%)	n(%)

Downloaded on 18 Jul 2025
System Version Number 1.0

Other	n(%)	n(%)	n(%)
Total Number of Major Protocol Deviations	n	n	n
Number of Subjects with Major Protocol Deviation	n(%)	n(%)	n(%)
Subject Missed Follow Up Visit	n(%)	n(%)	n(%)
…..	n(%)	n(%)	n(%)
Clinical Assessment Not Done	n(%)	n(%)	n(%)

Table 14.1.6 Summary of Device Deficiencies

ITT

	PureWick (N=xxx)	Hollister (N=xxx)	Total (N=xxx)
Total Number of Device Deficiencies	n	n	n
Total Number of Subjects with at least one Device Deficiency	n	n	n
Time of Device Deficiency			
Prior to Device Exposure	n/N (%)	n/N (%)	n/N (%)

Downloaded on 18 Jul 2025
System Version Number 1.0

During Device Exposure	n/N (%)	n/N (%)	n/N (%)
After Device Exposure	n/N (%)	n/N (%)	n/N (%)
Device Name/Identifier			
PureWick Female External Catheter	n/N (%)	n/N (%)	n/N (%)
PureWick Urine Collection System	n/N (%)	n/N (%)	n/N (%)
Hollister Female External Collection Pouch	n/N (%)	n/N (%)	n/N (%)
Failure Code			
Device Packaging Damaged/Broken	n/N (%)	n/N (%)	n/N (%)
Device Component Missing	n/N (%)	n/N (%)	n/N (%)
………	n/N (%)	n/N (%)	n/N (%)
………	n/N (%)	n/N (%)	n/N (%)
………	n/N (%)	n/N (%)	n/N (%)
Other	n/N (%)	n/N (%)	n/N (%)

Downloaded on 18 Jul 2025
System Version Number 1.0

Was Device Used to Treat Study Subject			
Yes	n/N (%)	n/N (%)	n/N (%)
No	n/N (%)	n/N (%)	n/N (%)
Was an Adverse Event Associated with Device Deficiency			
Yes	n/N (%)	n/N (%)	n/N (%)
No	n/N (%)	n/N (%)	n/N (%)
If NO, Could the Device Deficiency Have Led to a SADE if Suitable Action Had not been Taken, if the Intervention Had not been Made or if Circumstances Had been less Fortunate			
Yes	n/N (%)	n/N (%)	n/N (%)
No	n/N (%)	n/N (%)	n/N (%)
Did The Device Deficiency Meet the Definition of a Serious Health Threat?			
Yes	n/N (%)	n/N (%)	n/N (%)
No	n/N (%)	n/N (%)	n/N (%)

Programming Note: For all the % s, denominator is total number of Device Failures

Downloaded on 18 Jul 2025
System Version Number 1.0

Downloaded on 18 Jul 2025
System Version Number 1.0

Table 14.1.7 Treatment Summary

ITT

	PureWick (N = xx)	Hollister (N = xx)
Average Duration of Device Wear (hr)		
N	xxx	xxx
Mean (SD)	xx.x (xx.xx)	xx.x (xx.xx)
Median	xx.x	xx.x
Min – Max	xx - xx	xx - xx
Number of Days with a Void		
N	xxx	xxx
Mean (SD)	xx.x (xx.xx)	xx.x (xx.xx)
Median	xx.x	xx.x
Min – Max	xx - xx	xx - xx
Average of Nightly Canister (with Lid) Pre-Use Weight/ Average of Nightly Graduated Cylinder Pre-Use Weight (g)		

Downloaded on 18 Jul 2025
System Version Number 1.0

N	xxx	xxx
Mean (SD)	xx.x (xx.xx)	xx.x (xx.xx)
Median	xx.x	xx.x
Min – Max	xx - xx	xx - xx
Average of Nightly Canister (with Lid) Post-Use Weight/ Average of Nightly Graduated Cylinder Post-Use Weight (g)		
N	xxx	xxx
Mean (SD)	xx.x (xx.xx)	xx.x (xx.xx)
Median	xx.x	xx.x
Min – Max	xx - xx	xx - xx
Average of Nightly Flex Wick Pre-Use Weight (g)/Urine Drainage Bag + Tubing in Canister (no Lid) Pre-Use Weight (g)		
N	xxx	xxx
Mean (SD)	xx.x (xx.xx)	xx.x (xx.xx)
Median	xx.x	xx.x
Min – Max	xx - xx	xx - xx

Downloaded on 18 Jul 2025
System Version Number 1.0

Average of Nightly Flex Wick Post-Use Weight (g) /Urine Drainage Bag + Tubing in Canister (no Lid) Post-Use Weight (g)		
N	xxx	xxx
Mean (SD)	xx.x (xx.xx)	xx.x (xx.xx)
Median	xx.x	xx.x
Min – Max	xx - xx	xx - xx
Average of Nightly Bed Pad 1 Pre-Use Weight (g)		
N	xxx	xxx
Mean (SD)	xx.x (xx.xx)	xx.x (xx.xx)
Median	xx.x	xx.x
Min – Max	xx - xx	xx - xx
Average of Nightly Bed Pad 1 Post-Use Weight (g)		
N	xxx	xxx
Mean (SD)	xx.x (xx.xx)	xx.x (xx.xx)
Median	xx.x	xx.x
Min – Max	xx - xx	xx - xx

Downloaded on 18 Jul 2025
System Version Number 1.0

Number of Days Using 2nd Bed Pad		
N	xxx	xxx
Mean (SD)	xx.x (xx.xx)	xx.x (xx.xx)
Median	xx.x	xx.x
Min – Max	xx - xx	xx - xx
N	xxx	xxx
Average of Nightly Bed Pad 2 Pre-Use Weight (g)		
N	xxx	xxx
Mean (SD)	xx.x (xx.xx)	xx.x (xx.xx)
Median	xx.x	xx.x
Min – Max	xx - xx	xx - xx
Average of Nightly Bed Pad 2 Post-Use Weight (g)		
N	xxx	xxx
Mean (SD)	xx.x (xx.xx)	xx.x (xx.xx)
Median	xx.x	xx.x

Downloaded on 18 Jul 2025
System Version Number 1.0

Min – Max	xx - xx	xx - xx
Number of Days Using the Mesh Underwear Provided		
N	xxx	
Mean (SD)	xx.x (xx.xx)	
Median	xx.x	
Min – Max	xx - xx	
N	xxx	
Average of Nightly Mesh Underwear Pre-Use Weight (g)		
N	xxx	
Mean (SD)	xx.x (xx.xx)	
Median	xx.x	
Min – Max	xx - xx	
Average of Nightly Mesh Underwear Post-Use Weight (g)		
N	xxx	
Mean (SD)	xx.x (xx.xx)	

Downloaded on 18 Jul 2025
System Version Number 1.0

Median	xx.x	
Min – Max	xx - xx	
Number of Days Having Urine Spill During the Urine Measurement Procedure		
N	xxx	xxx
Mean (SD)	xx.x (xx.xx)	xx.x (xx.xx)
Median	xx.x	xx.x
Min – Max	xx - xx	xx - xx
Number of Days Having Assistance Placing the Device		
N	xxx	xxx
Mean (SD)	xx.x (xx.xx)	xx.x (xx.xx)
Median	xx.x	xx.x
Min – Max	xx - xx	xx - xx
Number of Days Professional Caregiver Assisted the Placement		
N	xxx	xxx
Mean (SD)	xx.x (xx.xx)	xx.x (xx.xx)

Downloaded on 18 Jul 2025
System Version Number 1.0

Median	xx.x	xx.x
Min – Max	xx - xx	xx - xx
Number of Days Family Member Assisted the Placement		
N	xxx	xxx
Mean (SD)	xx.x (xx.xx)	xx.x (xx.xx)
Median	xx.x	xx.x
Min – Max	xx - xx	xx - xx
Number of Days Other People Assisted the Placement		
N	xxx	xxx
Mean (SD)	xx.x (xx.xx)	xx.x (xx.xx)
Median	xx.x	xx.x
Min – Max	xx - xx	xx - xx

Table 14.1.8 Void Summary

ITT

Downloaded on 18 Jul 2025
System Version Number 1.0

	PureWick (N = xx)	Hollister (N = xx)
Average of Nightly Captured Weight (g)		
N	xxx	xxx
Mean (SD)	xx.x (xx.xx)	xx.x (xx.xx)
Median	xx.x	xx.x
Min – Max	xx - xx	xx - xx
Average of Nightly Leaked Weight (g)		
N	xxx	xxx
Mean (SD)	xx.x (xx.xx)	xx.x (xx.xx)
Median	xx.x	xx.x
Min – Max	xx - xx	xx - xx
Average of Nightly Void Weight (g)		
N	xxx	xxx
Mean (SD)	xx.x (xx.xx)	xx.x (xx.xx)
Median	xx.x	xx.x

Downloaded on 18 Jul 2025
System Version Number 1.0

Min – Max	xx - xx	xx - xx

Table 14.2.1 Capture Rate Summary

ITT

Average of Nightly Capture Rate (%)	PureWick (N = xx)	Hollister (N = xx)
N	xxx	xxx
Mean (SD)	xx.x (xx.xx)	xx.x (xx.xx)
Median	xx.x	xx.x
Min – Max	xx - xx	xx - xx

Footnote:

[1] Analysis was performed using evaluable voids.

Downloaded on 18 Jul 2025
System Version Number 1.0

Table 14.2.1p Capture Rate Summary

PP

Footnote:

[1] Analysis was performed using evaluable voids.

Downloaded on 18 Jul 2025
System Version Number 1.0

Table 14.2.2.1 Non-inferiority Test of Capture Rate -Hypothesis Test Step 1

ITT

	Mean Difference	SD	One-sided 97.5% CI Lower Bound	P Value
Difference in Average of Nightly Capture Rate (%),PureWick Compared with Hollister	xx.x	xx.x	xx.x	0.xxx

Footnote:

[1] Analysis was performed using evaluable voids.

[2] One-sided p-value is provided by a two-sample t-test comparing the mean difference to -10, p-value<0.025 indicates rejection of the null hypothesis.

```
proc ttest data=sample sides=U alpha=0.025 h0=-10;
    class group;
    var value;
run;
```

Table 14.2.2.1p Non-inferiority Test of Capture Rate

PP

Downloaded on 18 Jul 2025
System Version Number 1.0

	Mean	SD	One-sided 97.5% CI Lower Bound	P Value
Difference in Average of Nightly Capture Rate (%),PureWick Compared with Hollister	xx.x	xx.x	xx.x	0.xxx

Footnote:

[1] Analysis was performed using evaluable voids.

[2] One-sided p-value is provided by a two-sample t-test comparing the mean difference to -10.

```
proc ttest data=sample sides=U alpha=0.025 h0=-10;
    class group;
    var value;
run;
```

Downloaded on 18 Jul 2025
System Version Number 1.0

Table 14.2.2.2 Mixed-effects Model of Capture Rate

ITT

	LS Mean	95% CI
Difference in Capture Rate (%),PureWick Compared with Hollister	xx.x	(xx.x , xx.x)

Footnote:

[1] Analysis was performed using evaluable voids.

[2] Treatment (device) and day were included as fixed effects, and subject was included as random effect.

```
proc mixed data = test_model;
class id device;
model cap_rate = device day/solution;
random id;
lsmeans device/cl pdiff alpha = 0.05;
run;
```

Downloaded on 18 Jul 2025
System Version Number 1.0

Table 14.2.2.3 Beta Regression of Capture Rate

ITT

		Estimate	95% CI
Capture Rate (%)	PureWick	xx.x	(xx.x , xx.x)
	Hollister	xx.x	(xx.x , xx.x)
Odds Ratio	PureWick Compared with Hollister	xx.x	(xx.x , xx.x)

Footnote:

[1] Analysis was performed using evaluable voids.

[2] Treatment (device) and day were included as fixed effects, and subject was included as random effect.

```
data test;
set adef;
aval2=(aval/100*(n-1)+0.5)/n;
run;
*n is the number of observations
proc glimmix data=test method=RSPL plots=studentpanel;
```

Downloaded on 18 Jul 2025
System Version Number 1.0

```
class  subjid trta ;
model aval2 = trta daynum/oddsratio dist=beta link=logit solution;
random intercept / subject=subjid solution;
estimate 'Purewick'  intercept 1
                       trta 1 0  /ILINK CL;

estimate 'Hollisetr'  intercept 1
                       trta 0 1  /ILINK CL;


run;
```

Table 14.2.3.1 Draize Scale for Skin Irritation Summary

ITT

Average of Daily Draize Scale	PureWick	Hollister
Total score		
N		
Mean (SD)		
Median		
Min – Max		

Downloaded on 18 Jul 2025
System Version Number 1.0

Erythema (redness) score		
N		
Mean (SD)		
Median		
Min – Max		
Edema (swelling) score		
N		
Mean (SD)		
Median		
Min – Max		
Bleeding score		
N		
Mean (SD)		
Median		
Min – Max		

Downloaded on 18 Jul 2025
System Version Number 1.0

Table 14.2.3.1p Draize Scale for Skin Irritation Summary

PP

Table 14.2.3.2 Non-inferiority Test of Draize Scale for Skin Irritation-Hypothesis Test Step 1

ITT

	Mean Difference	SD	One-sided 97.5% CI Upper Bound	P Value
Difference in Average of Daily Draize Scale Total Score, PureWick Compared with Hollister	xx.x	xx.x	xx.x	0.xxx

Footnote:

[1] One-sided p-value is provided by a two-sample t-test comparing the mean difference to 1.2, p-value<0.025 indicates rejection of the null hypothesis.

```
proc ttest data=sample sides=L alpha=0.025 h0=1.2;
    class group;
    var value;
run;
```

Table 14.2.3.2p Non-inferiority Test of Draize Scale for Skin Irritation

PP

Downloaded on 18 Jul 2025
System Version Number 1.0

	Mean Difference	SD	One-sided 97.5% CI Upper Bound	P Value
Difference in Average of Daily Draize Scale Total Score, PureWick Compared with Hollister	xx.x	xx.x	xx.x	0.xxx

Footnote:

[1] One-sided p-value is provided by a two-sample t-test comparing the mean difference to 1.2.

Table 14.2.3.3 Mixed Effects Model of Draize Scale for Skin Irritation

ITT

	Estimate	95% CI
Ratio of Daily Draize Scale Total Score, PureWick Compared with Hollister	xx.x	(xx.x , xx.x)
Odds Ratio of Skin Irritation, PureWick Compared with Hollister	xx.x	(xx.x , xx.x)

Footnote: Poisson model or Negative Binomial or zero-inflated Poisson model will be used to model the Draize scale data based on model fitness for the data. If Poisson model or Negative Binomial model is chosen for the analysis, then the odds ratio row in the table will not be available.

```
/* Poisson Regression */
proc glimmix data=draize_data;
    class group(reference='1') id;
    model value = group time  / dist=poisson link=log solution;
```

```
    random intercept / subject=id;
run;
Or
/* Negative Binomial Regression */

proc glimmix data=draize_data method=quad;
    class group(reference='1') id;
    model value = group time  / dist=negbin link=log solution;
    random intercept / subject=id;
run;

Or

/* Zero-Inflated Poisson Regression */
proc nlmixed data=draize_data;
   parms beta0=0 beta1=0 beta2=0  gamma0=0 gamma1=0 gamma2=0 sigma=1;

   /* Define the linear predictors */
   eta = beta0 + beta1*group + beta2*time +u;
   mu = exp(eta);

   /* Zero-inflation part */
   p = exp(gamma0 + gamma1*group+ gamma2*time+u) / (1 + exp(gamma0 + gamma1*group+ gamma2*time+u));

   /* Likelihood for zero-inflated Poisson */
   if value = 0 then
      ll = log(p + (1 - p) * exp(-mu));
   else
      ll = log(1 - p) + value*log(mu) - mu - lgamma(value + 1);

   /* Random effect distribution */
   model value ~ general(ll);
   random u ~ normal(0, sigma**2) subject=id;
```

Downloaded on 18 Jul 2025
System Version Number 1.0

```
run;
```

Table 14.2.4.1 Superiority Test of Capture Rate – Hypothesis Test Step 2

ITT

	Mean Difference	SD	One-sided 97.5% CI Lower Bound	P Value
Difference in Average of Nightly Capture Rate (%),PureWick Compared with Hollister	xx.x	xx.x	xx.x	0.xxx

Footnote:

[1] Analysis was performed using evaluable voids.

[2] One-sided p-value is provided by a two-sample t-test comparing the mean difference to 0.

```
proc ttest data=sample sides=U alpha=0.025 h0=0;
    class group;
    var value;
run;
```

Downloaded on 18 Jul 2025
System Version Number 1.0

Table 14.2.5.1 Tolerability - Number of Days of Actual Use Summary

ITT

Tolerability - Number of Days of Actual Use	PureWick (N = xx)	Hollister (N = xx)
N		
Mean (SD)		
Median		
Min – Max		

Table 14.2.5.2 Superiority Test of Tolerability (Number of Days of Actual Use) – Hypothesis Test Step 3

ITT

	Mean Difference	SD	One-sided 97.5% CI Lower Bound	P Value
Difference in Tolerability - Number of Days of Actual Use,PureWick Compared with Hollister	xx.x	xx.x	xx.x	0.xxx

Downloaded on 18 Jul 2025
System Version Number 1.0

Footnote:

[1] One-sided p-value is provided by a two-sample t-test comparing the mean difference to 0.

```
proc ttest data=sample sides=U alpha=0.025 h0=0;
    class group;
    var value;
run;
```

Table 14.2.6.1 Participant Ease of Use Questionnaire Summary

ITT

	PureWick (N = xx)	Hollister (N = xx)
How easy was the setup and placement of the device?		
1-Very Difficult	n/N(%)	n/N(%)
2-Somewhat Difficult	n/N(%)	n/N(%)
3-Average Difficulty	n/N(%)	n/N(%)
4-Somewhat Easy	n/N(%)	n/N(%)
5-Very Easy	n/N(%)	n/N(%)
Mean(SD)		
Median		

Downloaded on 18 Jul 2025
System Version Number 1.0

Min-Max		
How easy was the removal of the device?		
1-Very Difficult	n/N(%)	n/N(%)
2-Somewhat Difficult	n/N(%)	n/N(%)
3-Average Difficulty	n/N(%)	n/N(%)
4-Somewhat Easy	n/N(%)	n/N(%)
5-Very Easy	n/N(%)	n/N(%)
Mean(SD)		
Median		

Table 14.2.6.2 Superiority Test of Participant Ease of Use Questionnaire (PureWick Compared with Hollister) – Hypothesis Test Step 4

ITT

	Mean Difference	SD	One-sided 97.5% CI Lower Bound	P Value
Difference in Ease of Device Placement – Ease of Use Score	xx.x	xx.x	xx.x	0.xxx
Difference in Ease of Device Removal– Ease of Use Score	xx.x	xx.x	xx.x	0.xxx

[1] One-sided p-value is provided by a two-sample t-test comparing the mean difference to 0 for each test.

a. If the larger p-value is less than 0.01875, superiority for both tests will be claimed. Otherwise,

b. if the smaller p-value is less than 0.0125, superiority for the test with the smaller p-value will be claimed. Otherwise,

c. superiority will not be claimed.

```
proc ttest data=sample sides=U alpha=0.025 h0=0;
    by paramn;
    class group;
    var value;
run;
```

Table 14.2.7.1 Participant Comfort Questionnaire Summary

ITT

	PureWick (N = xx)	Hollister (N = xx)
How comfortable was the placement of the device?		
1-Very uncomfortable	n/N(%)	n/N(%)
2-Uncomfortable	n/N(%)	n/N(%)
3-Neither Comfortable nor Uncomfortable	n/N(%)	n/N(%)
4-Comfortable	n/N(%)	n/N(%)
5-Very Comfortable	n/N(%)	n/N(%)
Mean(SD)		
Median		

Downloaded on 18 Jul 2025
System Version Number 1.0

Min-Max		
How comfortable was the device during sleep?		
1-Very uncomfortable	n/N(%)	n/N(%)
2-Uncomfortable	n/N(%)	n/N(%)
3-Neither Comfortable nor Uncomfortable	n/N(%)	n/N(%)
4-Comfortable	n/N(%)	n/N(%)
5-Very Comfortable	n/N(%)	n/N(%)
Mean(SD)		
Median		
Min-Max		
How comfortable was the removal of the device?		
1-Very uncomfortable	n/N(%)	n/N(%)
2-Uncomfortable	n/N(%)	n/N(%)
3-Neither Comfortable nor Uncomfortable	n/N(%)	n/N(%)
4-Comfortable	n/N(%)	n/N(%)
5-Very Comfortable	n/N(%)	n/N(%)
Mean(SD)		
Median		
Min-Max		

Downloaded on 18 Jul 2025
System Version Number 1.0

Table 14.2.7.2 Superiority Test of Participant Comfort Questionnaire (PureWick Compared with Hollister) – Hypothesis Test Step 5

ITT

	Mean Difference	SD	One-sided 97.5% CI Lower Bound	P Value
Difference in Comfort of Device Placement – Comfort of Use Score	xx.x	xx.x	xx.x	0.xxx
Difference in Comfort of Device During Sleep – Comfort of Use Score	xx.x	xx.x	xx.x	0.xxx
Difference in Comfort of Device Removal– Comfort of Use Score	xx.x	xx.x	xx.x	0.xxx

[1] One-sided p-value is provided by a two-sample t-test comparing the mean difference to 0 for each test.

Suppose the retained alpha (one-sided) from the previous step is αr, when using Hochberg procedure, the three alpha levels for the one-sided tests are:

1. α1 = αr/3
2. α2 = αr/2
3. α3 = αr

a. If the largest p-value is less than α3, superiority for all three tests will be claimed, otherwise

b. if the second largest p-value is less than α2, superiority for the two tests with smaller p-values will be claimed, otherwise

c. if the smallest p-value is less than α1, superiority for the smallest test will be claimed, otherwise

d. superiority will not be claimed for any of the three tests.

```
proc ttest data=sample sides=U alpha=0.025 h0=0;
```

Downloaded on 18 Jul 2025
System Version Number 1.0

```
    by paramn;
    class group;
    var value;
run;
```

Table 14.2.8 End of Study Questionnaire Summary

ITT

	PureWick (N = xx)	Hollister (N = xx)
Would you like to continue using the urine collection device beyond this study		
Yes	n/N(%)	n/N(%)
No	n/N(%)	n/N(%)
How would you say your quality of life as it relates to urine management has changed compared to how it was while using your previous urine management solution?		
1-My quality of life has gotten worse	n/N(%)	n/N(%)
2-My quality of life has stayed about the same	n/N(%)	n/N(%)
3-My quality of life has gotten better	n/N(%)	n/N(%)
Mean(SD)	xx.x (xx.xx)	xx.x (xx.xx)
Median	xx.x	xx.x
Min-Max	xx - xx	xx - xx

Downloaded on 18 Jul 2025
System Version Number 1.0

How likely would you be to recommend the device to one of your loved ones?		
1-Very Unlikely	n/N(%)	n/N(%)
2-Unlikely	n/N(%)	n/N(%)
3-Neither likely Nor Unlikely	n/N(%)	n/N(%)
4-Likely	n/N(%)	n/N(%)
5-Very Likely	n/N(%)	n/N(%)
Mean(SD)	xx.x (xx.xx)	xx.x (xx.xx)
Median	xx.x	xx.x
Min-Max	xx - xx	xx - xx
How would you describe the dryness experienced with the device during sleep?		
1- Never Dry (Unacceptable)	n/N(%)	n/N(%)
2-Sometimes Dry (Unacceptable)	n/N(%)	n/N(%)
3-Usually Dry (Acceptable)	n/N(%)	n/N(%)
4-Always Dry to Mostly Always Dry (Acceptable)	n/N(%)	n/N(%)
Mean(SD)	xx.x (xx.xx)	xx.x (xx.xx)
Median	xx.x	xx.x
Min-Max	xx - xx	xx - xx

Downloaded on 18 Jul 2025
System Version Number 1.0

Template GFM-10018B

Table 14.2.9 Nocturia Quality of Life (N-QoL) Questionnaire Summary

ITT

Nocturia Quality of Life (N-QoL) Questionnaire	Baseline		Device Use Night 14		End of Treatment	
	PureWick (N = xx)	Hollister (N = xx)	PureWick (N = xx)	Hollister (N = xx)	PureWick (N = xx)	Hollister (N = xx)
Transformed total score						
N	xxx	xxx	xxx	xxx	xxx	xxx
Mean (SD)	xx.x (xx.xx)	xx.x (xx.xx)	xx.x (xx.xx)	xx.x (xx.xx)	xx.x (xx.xx)	xx.x (xx.xx)
Median	xx.x	xx.x	xx.x	xx.x	xx.x	xx.x
Min – Max	xx - xx	xx - xx	xx - xx	xx - xx	xx - xx	xx - xx
Transformed total score change from baseline						
N		xxx	xxx	xxx	xxx	xxx
Mean (SD)		xx.x (xx.xx)	xx.x (xx.xx)	xx.x (xx.xx)	xx.x (xx.xx)	xx.x (xx.xx)
Median		xx.x	xx.x	xx.x	xx.x	xx.x
Min – Max		xx - xx	xx - xx	xx - xx	xx - xx	xx - xx
Raw total score						

Downloaded on 18 Jul 2025
System Version Number 1.0

N	xxx	xxx	xxx	xxx	xxx	xxx
Mean (SD)	xx.x (xx.xx)	xx.x (xx.xx)	xx.x (xx.xx)	xx.x (xx.xx)	xx.x (xx.xx)	xx.x (xx.xx)
Median	xx.x	xx.x	xx.x	xx.x	xx.x	xx.x
Min – Max	xx - xx	xx - xx	xx - xx	xx - xx	xx - xx	xx - xx
Sleep/energy domain transformed total score						
N	xxx	xxx	xxx	xxx	xxx	xxx
Mean (SD)	xx.x (xx.xx)	xx.x (xx.xx)	xx.x (xx.xx)	xx.x (xx.xx)	xx.x (xx.xx)	xx.x (xx.xx)
Median	xx.x	xx.x	xx.x	xx.x	xx.x	xx.x
Min – Max	xx - xx	xx - xx	xx - xx	xx - xx	xx - xx	xx - xx
Sleep/energy domain transformed total score change from baseline						
N		xxx	xxx	xxx	xxx	xxx
Mean (SD)		xx.x (xx.xx)	xx.x (xx.xx)	xx.x (xx.xx)	xx.x (xx.xx)	xx.x (xx.xx)
Median		xx.x	xx.x	xx.x	xx.x	xx.x
Min – Max		xx - xx	xx - xx	xx - xx	xx - xx	xx - xx

Downloaded on 18 Jul 2025
System Version Number 1.0

Sleep/energy domain raw total score						
N	xxx	xxx	xxx	xxx	xxx	xxx
Mean (SD)	xx.x (xx.xx)	xx.x (xx.xx)	xx.x (xx.xx)	xx.x (xx.xx)	xx.x (xx.xx)	xx.x (xx.xx)
Median	xx.x	xx.x	xx.x	xx.x	xx.x	xx.x
Min – Max	xx - xx	xx - xx	xx - xx	xx - xx	xx - xx	xx - xx
Bother/concern domain transformed total score						
N	xxx	xxx	xxx	xxx	xxx	xxx
Mean (SD)	xx.x (xx.xx)	xx.x (xx.xx)	xx.x (xx.xx)	xx.x (xx.xx)	xx.x (xx.xx)	xx.x (xx.xx)
Median	xx.x	xx.x	xx.x	xx.x	xx.x	xx.x
Min – Max	xx - xx	xx - xx	xx - xx	xx - xx	xx - xx	xx - xx
Bother/concern domain transformed total score change from baseline						
N		xxx	xxx	xxx	xxx	xxx
Mean (SD)		xx.x (xx.xx)	xx.x (xx.xx)	xx.x (xx.xx)	xx.x (xx.xx)	xx.x (xx.xx)
Median		xx.x	xx.x	xx.x	xx.x	xx.x

Downloaded on 18 Jul 2025
System Version Number 1.0

Min – Max		xx - xx	xx - xx	xx - xx	xx - xx	xx - xx
Bother/concern domain raw total score						
N	xxx	xxx	xxx	xxx	xxx	xxx
Mean (SD)	xx.x (xx.xx)	xx.x (xx.xx)	xx.x (xx.xx)	xx.x (xx.xx)	xx.x (xx.xx)	xx.x (xx.xx)
Median	xx.x	xx.x	xx.x	xx.x	xx.x	xx.x
Min – Max	xx - xx	xx - xx	xx - xx	xx - xx	xx - xx	xx - xx
**Over the past 2 weeks, having to get up at night to urinate...**						
1.Has made it difficult for me to concentrate the next day						
N	xxx	xxx	xxx	xxx	xxx	xxx
Mean (SD)	xx.x (xx.xx)	xx.x (xx.xx)	xx.x (xx.xx)	xx.x (xx.xx)	xx.x (xx.xx)	xx.x (xx.xx)
Median	xx.x	xx.x	xx.x	xx.x	xx.x	xx.x
Min – Max	xx - xx	xx - xx	xx - xx	xx - xx	xx - xx	xx - xx
2.Has made me feel generally low in energy the next day						

Downloaded on 18 Jul 2025
System Version Number 1.0

N	xxx	xxx	xxx	xxx	xxx	xxx
Mean (SD)	xx.x (xx.xx)	xx.x (xx.xx)	xx.x (xx.xx)	xx.x (xx.xx)	xx.x (xx.xx)	xx.x (xx.xx)
Median	xx.x	xx.x	xx.x	xx.x	xx.x	xx.x
Min – Max	xx - xx	xx - xx	xx - xx	xx - xx	xx - xx	xx - xx
3.Has required me to nap during the day						
N	xxx	xxx	xxx	xxx	xxx	xxx
Mean (SD)	xx.x (xx.xx)	xx.x (xx.xx)	xx.x (xx.xx)	xx.x (xx.xx)	xx.x (xx.xx)	xx.x (xx.xx)
Median	xx.x	xx.x	xx.x	xx.x	xx.x	xx.x
Min – Max	xx - xx	xx - xx	xx - xx	xx - xx	xx - xx	xx - xx
4.Has made me less productive the next day						
N	xxx	xxx	xxx	xxx	xxx	xxx
Mean (SD)	xx.x (xx.xx)	xx.x (xx.xx)	xx.x (xx.xx)	xx.x (xx.xx)	xx.x (xx.xx)	xx.x (xx.xx)
Median	xx.x	xx.x	xx.x	xx.x	xx.x	xx.x
Min – Max	xx - xx	xx - xx	xx - xx	xx - xx	xx - xx	xx - xx

Downloaded on 18 Jul 2025
System Version Number 1.0

5.Has caused me to participate less in activities I enjoy						
N	xxx	xxx	xxx	xxx	xxx	xxx
Mean (SD)	xx.x (xx.xx)	xx.x (xx.xx)	xx.x (xx.xx)	xx.x (xx.xx)	xx.x (xx.xx)	xx.x (xx.xx)
Median	xx.x	xx.x	xx.x	xx.x	xx.x	xx.x
Min – Max	xx - xx	xx - xx	xx - xx	xx - xx	xx - xx	xx - xx
6.Has caused me to be careful about when or how much I drink						
N	xxx	xxx	xxx	xxx	xxx	xxx
Mean (SD)	xx.x (xx.xx)	xx.x (xx.xx)	xx.x (xx.xx)	xx.x (xx.xx)	xx.x (xx.xx)	xx.x (xx.xx)
Median	xx.x	xx.x	xx.x	xx.x	xx.x	xx.x
Min – Max	xx - xx	xx - xx	xx - xx	xx - xx	xx - xx	xx - xx
7.Has made it difficult for me to get enough sleep at night						
N	xxx	xxx	xxx	xxx	xxx	xxx
Mean (SD)	xx.x (xx.xx)	xx.x (xx.xx)	xx.x (xx.xx)	xx.x (xx.xx)	xx.x (xx.xx)	xx.x (xx.xx)
Median	xx.x	xx.x	xx.x	xx.x	xx.x	xx.x

Downloaded on 18 Jul 2025
System Version Number 1.0

Min – Max	xx - xx	xx - xx	xx - xx	xx - xx	xx - xx	xx - xx
**Over the past 2 weeks, I have been...**						
8.Concerned that I am disturbing others in the house because of having to get up at night to urinate						
N	xxx	xxx	xxx	xxx	xxx	xxx
Mean (SD)	xx.x (xx.xx)	xx.x (xx.xx)	xx.x (xx.xx)	xx.x (xx.xx)	xx.x (xx.xx)	xx.x (xx.xx)
Median	xx.x	xx.x	xx.x	xx.x	xx.x	xx.x
Min – Max	xx - xx	xx - xx	xx - xx	xx - xx	xx - xx	xx - xx
9.Preoccupied about having to get up at night to urinate						
N	xxx	xxx	xxx	xxx	xxx	xxx
Mean (SD)	xx.x (xx.xx)	xx.x (xx.xx)	xx.x (xx.xx)	xx.x (xx.xx)	xx.x (xx.xx)	xx.x (xx.xx)
Median	xx.x	xx.x	xx.x	xx.x	xx.x	xx.x
Min – Max	xx - xx	xx - xx	xx - xx	xx - xx	xx - xx	xx - xx

Downloaded on 18 Jul 2025
System Version Number 1.0

10.Worried that this condition will get worse in the future						
N	xxx	xxx	xxx	xxx	xxx	xxx
Mean (SD)	xx.x (xx.xx)	xx.x (xx.xx)	xx.x (xx.xx)	xx.x (xx.xx)	xx.x (xx.xx)	xx.x (xx.xx)
Median	xx.x	xx.x	xx.x	xx.x	xx.x	xx.x
Min – Max	xx - xx	xx - xx	xx - xx	xx - xx	xx - xx	xx - xx
11.Worried that there is no effective treatment for this condition (having to get up at night to urinate)						
N	xxx	xxx	xxx	xxx	xxx	xxx
Mean (SD)	xx.x (xx.xx)	xx.x (xx.xx)	xx.x (xx.xx)	xx.x (xx.xx)	xx.x (xx.xx)	xx.x (xx.xx)
Median	xx.x	xx.x	xx.x	xx.x	xx.x	xx.x
Min – Max	xx - xx	xx - xx	xx - xx	xx - xx	xx - xx	xx - xx
12.Overall, how bothersome has having to get up at night to urinate been during the past 2 weeks?						
N	xxx	xxx	xxx	xxx	xxx	xxx
Mean (SD)	xx.x (xx.xx)	xx.x (xx.xx)	xx.x (xx.xx)	xx.x (xx.xx)	xx.x (xx.xx)	xx.x (xx.xx)

Downloaded on 18 Jul 2025
System Version Number 1.0

Median	xx.x	xx.x	xx.x	xx.x	xx.x	xx.x
Min – Max	xx - xx	xx - xx	xx - xx	xx - xx	xx - xx	xx - xx
13.Overall I would rate my quality of life to be						
N	xxx	xxx	xxx	xxx	xxx	xxx
Mean (SD)	xx.x (xx.xx)	xx.x (xx.xx)	xx.x (xx.xx)	xx.x (xx.xx)	xx.x (xx.xx)	xx.x (xx.xx)
Median	xx.x	xx.x	xx.x	xx.x	xx.x	xx.x
Min – Max	xx - xx	xx - xx	xx - xx	xx - xx	xx - xx	xx - xx

Table 14.2.10.1 PROMIS Sleep Disturbance Questionnaire - PureWick

ITT

PROMIS Sleep Disturbance Score	Baseline	Device Use Night 7	Device Use Night 14	Device Use Night 21	End of Treatment
Total T-score					
N	xxx	xxx	xxx	xxx	xxx
Mean (SD)	xx.x (xx.xx)	xx.x (xx.xx)	xx.x (xx.xx)	xx.x (xx.xx)	xx.x (xx.xx)

Downloaded on 18 Jul 2025
System Version Number 1.0

Median	xx.x	xx.x	xx.x	xx.x	xx.x
Min – Max	xx - xx	xx - xx	xx - xx	xx - xx	xx - xx
Total T-score change from Baseline					
N		xxx	xxx	xxx	xxx
Mean (SD)		xx.x (xx.xx)	xx.x (xx.xx)	xx.x (xx.xx)	xx.x (xx.xx)
Median		xx.x	xx.x	xx.x	xx.x
Min – Max		xx - xx	xx - xx	xx - xx	xx - xx
Total raw score					
N	xxx	xxx	xxx	xxx	xxx
Mean (SD)	xx.x (xx.xx)	xx.x (xx.xx)	xx.x (xx.xx)	xx.x (xx.xx)	xx.x (xx.xx)
Median	xx.x	xx.x	xx.x	xx.x	xx.x
Min – Max	xx - xx	xx - xx	xx - xx	xx - xx	xx - xx
**My sleep quality was**					
5-Very Poor	n/N(%)	n/N(%)	n/N(%)	n/N(%)	n/N(%)
4-Poor	n/N(%)	n/N(%)	n/N(%)	n/N(%)	n/N(%)

Downloaded on 18 Jul 2025
System Version Number 1.0

3-Fair	n/N(%)	n/N(%)	n/N(%)	n/N(%)	n/N(%)
2-Good	n/N(%)	n/N(%)	n/N(%)	n/N(%)	n/N(%)
1-Very Good	n/N(%)	n/N(%)	n/N(%)	n/N(%)	n/N(%)
Mean (SD)	xx.x (xx.xx)	xx.x (xx.xx)	xx.x (xx.xx)	xx.x (xx.xx)	xx.x (xx.xx)
Median	xx.x	xx.x	xx.x	xx.x	xx.x
Min – Max	xx - xx	xx - xx	xx - xx	xx - xx	xx - xx
**My sleep was refreshing**					
5-Not at All	n/N(%)	n/N(%)	n/N(%)	n/N(%)	n/N(%)
4-A Little Bit	n/N(%)	n/N(%)	n/N(%)	n/N(%)	n/N(%)
3-Somewhat	n/N(%)	n/N(%)	n/N(%)	n/N(%)	n/N(%)
2-Quite a Bit	n/N(%)	n/N(%)	n/N(%)	n/N(%)	n/N(%)
1-Very Much	n/N(%)	n/N(%)	n/N(%)	n/N(%)	n/N(%)
Mean (SD)	xx.x (xx.xx)	xx.x (xx.xx)	xx.x (xx.xx)	xx.x (xx.xx)	xx.x (xx.xx)
Median	xx.x	xx.x	xx.x	xx.x	xx.x
Min – Max	xx - xx	xx - xx	xx - xx	xx - xx	xx - xx

Downloaded on 18 Jul 2025
System Version Number 1.0

**I had a problem with my sleep**					
1-Not at All	n/N(%)	n/N(%)	n/N(%)	n/N(%)	n/N(%)
2-A Little Bit	n/N(%)	n/N(%)	n/N(%)	n/N(%)	n/N(%)
3-Somewhat	n/N(%)	n/N(%)	n/N(%)	n/N(%)	n/N(%)
4-Quite a Bit	n/N(%)	n/N(%)	n/N(%)	n/N(%)	n/N(%)
5-Very Much	n/N(%)	n/N(%)	n/N(%)	n/N(%)	n/N(%)
Mean (SD)	xx.x (xx.xx)	xx.x (xx.xx)	xx.x (xx.xx)	xx.x (xx.xx)	xx.x (xx.xx)
Median	xx.x	xx.x	xx.x	xx.x	xx.x
Min – Max	xx - xx	xx - xx	xx - xx	xx - xx	xx - xx
**I had difficulty falling asleep**					
1-Not at All	n/N(%)	n/N(%)	n/N(%)	n/N(%)	n/N(%)
2-A Little Bit	n/N(%)	n/N(%)	n/N(%)	n/N(%)	n/N(%)
3-Somewhat	n/N(%)	n/N(%)	n/N(%)	n/N(%)	n/N(%)
4-Quite a Bit	n/N(%)	n/N(%)	n/N(%)	n/N(%)	n/N(%)
5-Very Much	n/N(%)	n/N(%)	n/N(%)	n/N(%)	n/N(%)

Downloaded on 18 Jul 2025
System Version Number 1.0

Mean (SD)	xx.x (xx.xx)	xx.x (xx.xx)	xx.x (xx.xx)	xx.x (xx.xx)	xx.x (xx.xx)
Median	xx.x	xx.x	xx.x	xx.x	xx.x
Min – Max	xx - xx	xx - xx	xx - xx	xx - xx	xx - xx

Table 14.2.10.2 PROMIS Sleep Disturbance Questionnaire - Hollister

ITT

Downloaded on 18 Jul 2025
System Version Number 1.0

Table 14.3.1 Summary of Adverse Events

AT Subjects

	PureWick (N=xxx)		Hollister (N=xxx)	
	By Events	By Subjects	By Events	By Subjects
Any Adverse Events	xxx	n (xx.x%)	xxx	n (xx.x%)
Severity				
Mild	xxx	n (xx.x%)	xxx	n (xx.x%)
Moderate	xxx	n (xx.x%)	xxx	n (xx.x%)
Severe	xxx	n (xx.x%)	xxx	n (xx.x%)
Relationship to Study Device				
Causal	xxx	n (xx.x%)	xxx	n (xx.x%)
Probable	xxx	n (xx.x%)	xxx	n (xx.x%)
Possible	xxx	n (xx.x%)	xxx	n (xx.x%)

Downloaded on 18 Jul 2025
System Version Number 1.0

Not Related	xxx	n (xx.x%)	xxx	n (xx.x%)
Relationship to Procedure				
Causal	xxx	n (xx.x%)	xxx	n (xx.x%)
Probable	xxx	n (xx.x%)	xxx	n (xx.x%)
Possible	xxx	n (xx.x%)	xxx	n (xx.x%)
Not Related	xxx	n (xx.x%)	xxx	n (xx.x%)
Serious AE (SAE)	xxx	n (xx.x%)	xxx	n (xx.x%)
Relationship to Study Device				
Causal	xxx	n (xx.x%)	xxx	n (xx.x%)
Probable	xxx	n (xx.x%)	xxx	n (xx.x%)
Possible	xxx	n (xx.x%)	xxx	n (xx.x%)
Not Related	xxx	n (xx.x%)	xxx	n (xx.x%)
Relationship to Procedure				
Causal	xxx	n (xx.x%)	xxx	n (xx.x%)

Downloaded on 18 Jul 2025
System Version Number 1.0

Probable	xxx	n (xx.x%)	xxx	n (xx.x%)
Possible	xxx	n (xx.x%)	xxx	n (xx.x%)
Not Related	xxx	n (xx.x%)	xxx	n (xx.x%)
Death	xxx	n (xx.x%)	xxx	n (xx.x%)
UADE	xxx	n (xx.x%)	xxx	n (xx.x%)
Serious Health Threat	xxx	n (xx.x%)	xxx	n (xx.x%)

For by-subject summaries, subjects are counted in the highest severity or most-related category.

I Table 14.3.2 Subjects with Adverse Events by Body System and Preferred Term

AT Subjects

Body System<br>Preferred Term	PureWick<br>(N=xxx)	Hollister<br>(N=xxx)
Total no. of Events	n	n

Downloaded on 18 Jul 2025
System Version Number 1.0

Total no. of Subjects with at least one AE	n/N	n/N
	n/N	n/N
	n/N	n/N
	n/N	n/N
	n/N	n/N
	n/N	n/N
	n/N	n/N
	n/N	n/N

MedDRA dictionary version 27.1 will be used for coding.

Sort by body system organ.

Table 14.3.3 Subjects with Device Related Adverse Events by Body System and Preferred Term

AT Subjects

Body System<br>Preferred Term	PureWick<br>(N=xxx)	Hollister<br>(N=xxx)
Any Device Related AE	n	n

Subjects with any Procedure Related AE	n/N	n/N
	n/N	n/N
	n/N	n/N
	n/N	n/N
	n/N	n/N
	n/N	n/N
	n/N	n/N
….	n/N	n/N

Related includes Possible, Probable and Causal.

Table 14.3.4 Subjects with Procedure Related AE by Body System and Preferred Term

AT Subjects

Body System<br>Preferred Term	PureWick<br>(N=xxx)	Hollister<br>(N=xxx)
Any Procedure Related AE	n	n
Subjects with any Procedure Related AE	n/N	n/N
	n/N	n/N

Downloaded on 18 Jul 2025
System Version Number 1.0

	n/N	n/N
	n/N	n/N
	n/N	n/N
	n/N	n/N
	n/N	n/N
	n/N	n/N

Related includes Possible, Probable and Causal.

Downloaded on 18 Jul 2025
System Version Number 1.0

Table 14.3.5 Subjects with Serious Adverse Events by Body System and Preferred Term

AT Subjects

	PureWick (N=xxx)	Hollister (N=xxx)
Any Serious AE	n	n
Subjects with any SAE	n/N	n/N
	n/N	n/N
	n/N	n/N
	n/N	n/N
	n/N	n/N
	n/N	n/N
	n/N	n/N
	n/N	n/N

Downloaded on 18 Jul 2025
System Version Number 1.0

3.0 SHELLS AND SPECIFICATIONS FOR LISTINGS

Listing 16.1.1 Randomization

Enrolled

Subject ID	Date of Informed Consent	Consented Protocol Version	Did the subject meet all eligibility criteria?	Randomized?	Date of Randomization	Randomization No.
				Yes		
				No		

Listing 16.1.2 Subject Enrollment Status

Enrolled

Subject ID	What is the status of the subject?	Planned Treatment	Actual Treatment

Listing 16.1.3 Subjects with Screen Failures

Enrolled

Downloaded on 18 Jul 2025
System Version Number 1.0

Subject ID	Eligibility criteria not met

Listing 16.1.4 Disposition - End of Study

ITT

Subject ID	Planned Treatment	Date of Study Completion/Discontinuation	Status	Other, Specify	Date of Death	Event leading to discontinuation

Listing 16.1.5 Protocol Deviations

ITT

Subject ID	Planned Treatment	Date of Deviation	Nature of Deviation	Additional Details	Major Protocol Deviation

Downloaded on 18 Jul 2025
System Version Number 1.0

Programming note: Only list subjects who have protocol deviation.

Listing 16.1.6 Demographics

ITT

Subject ID	Planned Treatment	Age	Sex	Ethnicity	Race
				Not Hispanic or Latino	

Listing 16.1.7 Primary Diagnosis

ITT

Subject ID	Planned Treatment	Primary diagnosis	Other/Specify	Start date	Additional medical history to report
					Yes
					No

Listing 16.1.8 Medical History

ITT

Subject ID	Planned Treatment	Medical history event term	Start date	Ongoing?	Stop date

Downloaded on 18 Jul 2025
System Version Number 1.0

Subject ID	Planned Treatment	Medical history event term	Start date	Ongoing?	Stop date

Listing 16.1.9 Vital Signs

ITT

Subject ID	Planned Treatment	Age	Date	Height (cm)	Weight (kg)	BMI (kg/m^2)

Listing 16.1.10 Participant Self-Reported Skin Assessment

ITT

Subject ID	Planned Treatment	Date of Assessment	Any Skin Irritation

Downloaded on 18 Jul 2025
System Version Number 1.0

Listing 16.1.11.1.1 Treatment Details - PureWick Part 1
AT Subjects

Subject ID	Event Group	Date of device placement	Time of device placement	Date of device removal	Time of device removal	Did the Participant Have a Void?	Canister (with lid) pre-use weight (g)	Canister (with lid) post-use weight (g)	Flex wick pre-use weight (g)	Flex wick post-use weight (g)	Bed pad 1 pre-use weight (g)	Bed pad 1 post-use weight (g)
	Device Use Night 1											

Programming note: Only list subjects who have the device placed.

Listing 16.1.11.1.2 Treatment Details - PureWick Part 2
AT Subjects

Subject ID	Event Group	Was 2nd bed pad used?	Bed pad 2 pre-use weight (g)	Bed pad 2 post-use weight (g)	Did participant use the mesh underwear	Mesh underwear pre-use weight (g)	Mesh underwear post-use weight (g)	Did any urine spill during the urine measurement procedure?	Did you have assistance placing the device?	If Yes, please specify who assisted	If Other, please specify

Downloaded on 18 Jul 2025
System Version Number 1.0

					provided while using the study device?						
	Device Use Night 1										

Listing 16.1.11.2.1 Treatment Details – Hollister(R) Part 1
AT Subjects

Subject ID	Event Group	Date of device placement	Time of device placement	Date of device removal	Time of device removal	Did the Participant Have a Void?	Graduated cylinder pre-use weight (g)	Graduated cylinder post-use weight (g)	Urine drainage bag + tubing in canister (no lid) pre-use weight (g)	Urine drainage bag + tubing in canister (no lid) post-use weight (g)
	Device Use Night 1									

Programming note: Only list subjects who have the device placed.

Listing 16.1.11.2.2 Treatment Details - Hollister(R) Part 2
AT Subjects

Subject ID	Event Group	Bed pad 1 pre-use weight (g)	Bed pad 1 post-use weight (g)	Was 2nd bed pad used?	Bed pad 2 pre-use weight (g)	Bed pad 2 post-use weight (g)	Did any urine spill during the urine measurement procedure?	Did you have assistance placing the device?	If Yes, please specify who assisted	If Other, please specify
	Device Use Night 1									

Listing 16.1.12 Device Wear
ITT Subjects

Downloaded on 18 Jul 2025
System Version Number 1.0

Subject ID	Planned Treatment	Event Group	Did Participant wear device overnight?

Listing 16.2.1 Capture Rate
ITT

Subject ID	Planned Treatment	Event Group	Date of device placement	Leaked Weight (g)	Captured Weight (g)	Capture Rate (%)	Evaluable
		Device Use Night 1					Yes
							No

Listing 16.2.2 Draize Scale for Skin Irritation
ITT

Subject ID	Planned Treatment	Event Group	Date of assessment	Erythema (redness) score	Edema (swelling) score	Bleeding score

Downloaded on 18 Jul 2025
System Version Number 1.0

		Device Use Night 1				

Programming note: Only list subjects who have completed the questionnaire.

Listing 16.2.3.1 Nocturia Quality of Life (N-QoL) Questionnaire Part 1
ITT

Subject ID	Planned Treatment	Event Group		Over the past 2 weeks, having to get up at night to urinate...						
				1. Has made it difficult for me to concentrate the next day	2. Has made me feel generally low in energy the next day	3. Has required me to nap during the day	4. Has made me less productive the next day	5. Has caused me to participate less in activities I enjoy	6. Has caused me to be careful about when or how much I drink	7. Has made it difficult for me to get enough sleep at night

Downloaded on 18 Jul 2025
System Version Number 1.0

			Date questionnaire completed							
		Device Use Night 1								

Programming note: Only list subjects who have completed the questionnaire.

Listing 16.2.3.2 Nocturia Quality of Life (N-QoL) Questionnaire Part 2

ITT

			Over the past 2 weeks, I have been...	

Subject ID	Planned Treatment	Event Group	8. Concerned that I am disturbing others in the house because of having to get up at night to urinate	9. Preoccupied about having to get up at night to urinate	10. Worried that this condition will get worse in the future	11. Worried that there is no effective treatment for this condition (having to get up at night to urinate)	12. Overall, how bothersome has having to get up at night to urinate been during the past 2 weeks?	13. Overall quality of life
		Device Use Night 1						

Programming note: Only list subjects who have completed the questionnaire.

Listing 16.2.4 PROMIS Sleep Disturbance Questionnaire
ITT

Downloaded on 18 Jul 2025
System Version Number 1.0

Subject ID	Planned Treatment	Event Group	Date PROMIS sleep disturbance questionnaire completed	My sleep quality was	My sleep was refreshing	I had a problem with my sleep	I had difficulty falling asleep
		Device Use Night 7					

Programming note: Only list subjects who have completed the questionnaire.

Listing 16.2.5.1 PureWick Participant Comfort Questionnaire
ITT

Subject ID	How Comfortable was the Placement?/Comments	How Comfortable was the Device During Sleep? /Comments	How Comfortable was the Removal? /Comments
	2-Uncomfortable	1-Very Uncomfortable	5-Very Comfortable

Downloaded on 18 Jul 2025
System Version Number 1.0

Programming note: Only list subjects who have completed the questionnaire.

Listing 16.2.5.2 Hollister Participant Comfort Questionnaire
ITT

Subject ID	How Comfortable was the Placement?/Comments	How Comfortable was the Device During Sleep? /Comments	How Comfortable was the Removal? /Comments
	2-Uncomfortable	1-Very Uncomfortable	5-Very Comfortable

Programming note: Only list subjects who have completed the questionnaire.

Listing 16.2.6.1 PureWick-Participant Ease of Use Questionnaire
ITT

Subject ID	How Easy was the Setup and Placement of the Device?/Comments	How Easy was the Removal of the Device?/Comments

Downloaded on 18 Jul 2025
System Version Number 1.0

	1-Very Difficult	5-Very Easy

Programming note: Only list subjects who have completed the questionnaire.

Listing 16.2.6.2 Hollister-Participant Ease of Use Questionnaire
ITT

Subject ID	How Easy was the Setup and Placement of the Device?/Comments	How Easy was the Removal of the Device?/Comments
	1-Very Difficult	5-Very Easy

Programming note: Only list subjects who have completed the questionnaire.

Listing 16.2.7 End of Study Participant Survey
ITT

Downloaded on 18 Jul 2025
System Version Number 1.0

Subject ID	Planned Treatment	Would you like to continue using the urine collection device beyond this study? / Comment	How would you say your quality of life as it relates to urine management has changed compared to how it was while using your previous urine management solution?/Comment	How likely would you be to recommend the urine collection device to one of your loved ones? / Comment	How would you describe the dryness experienced with the urine collection device during sleep?/ Comments

Programming note: Only list subjects who have completed the survey.

Listing 16.2.8 Device Deficiencies

ITT

Subject ID	Date of Device Deficiencies	Time of Device Deficiencies	Device Name/identifier	Lot Number	Failure Code	Details	Additional Details	Device Used to Treat Subject?	AE Associated with Device Deficiencies?	Could The Device Deficiency Have Led to a SADE?	Serious Health Threat
	DDMMYY							Yes/No	Yes/No		

Programming note: Only list subjects who have device deficiency.

Listing 16.3.1 Adverse Events

AT Subjects

Downloaded on 18 Jul 2025
System Version Number 1.0

Subject ID	Actual Treatment	AE(Reported)/SOC/PT	Start Date / End Date	Outcome of AE	Severity	Relation to Study Device	Relation to Procedure	SAE	UADE	Serious Health Threat	Additional Details
									Yes/No		

Listing 16.3.2 Serious Adverse Events

AT Subjects

Subject ID	Actual Treatment	AE(Reported)/SOC/PT	Start Date	End Date	Serious Criteria	If Require or Prolongs Hospitalization	
						Admission Date	Discharge Date

4.0 FIGURES

Figure 1.1 Capture Rate over Time

Programming note: Scatter plot, Capture Rate (y axis) vs. Device Use Night # (x axis), with different colors representing PureWick and Hollister.

Figure 1.2 Draize Scale over Time

Programming note: Scatter plot, Draize Scale Total Score (y axis) vs. Device Use Night # (x axis), with different colors representing PureWick and Hollister.

Downloaded on 18 Jul 2025
System Version Number 1.0

Figure 1.3 N-QoL over Time

Programming note: Scatter plot, N-QoL Transformed Total Score (y axis) vs. Device Use Night # (x axis), with different colors representing PureWick and Hollister.

Figure 1.4 PROMIS Sleep Disturbance Score over Time

Programming note: Scatter plot, PROMIS Total T-score (y axis) vs. Device Use Night # (x axis), with different colors representing PureWick and Hollister.

Figure 2.1 Correlation between Average Capture Rate and N-QoL Weeks 1-2

Programming note: Scatter plot, N-QoL Transformed Total Score (y axis) vs. Capture rate (x axis), with different colors representing PureWick and Hollister, the average nightly capture rate for each subject will be computed and used as x axis. N-QoL data collected after device use night 14, 15 or 16 will be used as y axis. Capture rate is based on data collected for device use nights 1-5.

Figure 2.2 Correlation between Average Capture Rate and N-QoL Weeks 3-4

Programming note: Scatter plot, N-QoL Transformed Total Score (y axis) vs. Capture rate (x axis), with different colors representing PureWick and Hollister, the average nightly capture rate for each subject will be computed and used as x axis. N-QoL data collected at the end of treatment will be used as y axis. Capture rate is based on data collected for device use from night 24 and onward.

Figure 3.1 Correlation between Average Draize Scale and N-QoL Weeks 1-2

Programming note: Scatter plot, N-QoL Transformed Total Score vs. Draize Scale Total Score average per subject per device (x axis), with different colors representing PureWick and Hollister. N-QoL data collected after device use night 14, 15 or 16 will be used as y axis. Draize Scale is based on data collected for device use nights 1-5.

Figure 3.2 Correlation between Average Draize Scale and N-QoL Weeks 3-4

Programming note: Scatter plot, N-QoL Transformed Total Score vs. Draize Scale Total Score average per subject per device (x axis) for week 3-4, with different colors representing PureWick and Hollister. N-QoL data collected at the end of treatment will be used as y axis. Draize Scale is based on data collected for device use from night 24 and onward.

Downloaded on 18 Jul 2025
System Version Number 1.0

Figure 4.1 Correlation between Average Capture Rate and PROMIS Weeks 1

Programming note: Scatter plot, PROMIS Total T-score (y axis) vs. Capture rate (x axis), with different colors representing PureWick and Hollister, the average nightly capture rate for each subject will be computed and used as x axis. PROMIS data collected after device use nights 7,8 and 9 will be used as y axis. Capture rate is based on data collected for device use nights 1-5.

Figure 4.2 Correlation between Average Capture Rate and PROMIS Weeks 4

Programming note: Scatter plot, PROMIS Total T-score (y axis) vs. Capture rate (x axis), with different colors representing PureWick and Hollister, the average nightly capture rate for each subject will be computed and used as x axis. PROMIS data collected at the end of treatment will be used as y axis. Capture rate is based on data collected for device use from night 24 and onward.

Figure 5.1 Correlation between Average Draize Scale and PROMIS Weeks 1

Programming note: Scatter plot, PROMIS Total T-Score vs. Draize Scale Total Score average per subject per device (x axis), with different colors representing PureWick and Hollister. PROMIS Total T-Score data collected after device use nights 7,8 and 9 will be used as y axis. Draize Scale is based on data collected for device use nights 1-5.

Figure 5.2 Correlation between Average Draize Scale and PROMIS Weeks 4

Programming note: Scatter plot, PROMIS Total T-Score vs. Draize Scale Total Score average per subject per device (x axis), with different colors representing PureWick and Hollister. PROMIS Total T-Score data collected at the end of treatment will be used as y axis. Draize Scale is based on data collected for device use from night 24 and onward.

5.0 REFERENCES

None.

6.0 VERSION HISTORY

Vers. #	Date	Change Owner	Description of Change(s)
01	17-Jul-2025	Shuangshuang Fu	Original

Downloaded on 18 Jul 2025
System Version Number 1.0

Signature Page for VV-TMF-358750 v1.0

Reason for signing: Finalize	Name: Shuangshuang Fu<br>Role: Clinical Statistician<br>Date of signature: 17-Jul-2025 17:33:09 GMT+0000

Reason for signing: Finalize	Name: Yanchang Zhang<br>Role: Clinical Statistician<br>Date of signature: 17-Jul-2025 17:34:20 GMT+0000

Reason for signing: Finalize	Name: Danielle Redmond<br>Role: Medical Affairs Core Team Member<br>Date of signature: 17-Jul-2025 19:42:19 GMT+0000

Reason for signing: Finalize	Name: Swathi Vasireddi<br>Role: Clinical Statistical Programmer<br>Date of signature: 18-Jul-2025 18:02:33 GMT+0000

Reason for signing: Finalize	Name: SengDao VanMany<br>Role: Clinical Project Management<br>Date of signature: 18-Jul-2025 18:05:17 GMT+0000

Signature Page for VV-TMF-358750 v1.0

Downloaded on 18 Jul 2025
System Version Number 1.0
