# Supplementary material for: Safety, Efficacy, and Patient-Reported Outcomes of the PureWick™ System Versus Comparator for Nocturnal Urinary Incontinence in the Home Setting: Results of a Randomized Trial
Source: J Clin Med. 2025 Dec 9;14(24):8699. doi: 10.3390/jcm14248699 (PMC12734071; doi:10.3390/jcm14248699)
Supplement: Supplementary file 1 [file jcm-14-08699-s001.zip › S2_Measurement Anomalies.pdf]

Listing 16.1.5
Protocol Deviations
ITT

Subject ID	Planned Treatment	Date of Deviation	Nature of Deviation	Additional Details	Major Protocol Deviation
101002	PureWick	02DEC2024	Clinical Assessment Not Done	patient did not wear device	No
101004	Hollister	04JAN2025	Clinical Assessment Not Done	Nurse assigned did not complete Draize	No
	Hollister	05JAN2025	Clinical Assessment Not Done	Nurse assigned did not complete Draize assessment documentation.	No
101005	Hollister	10JAN2025	Clinical Assessment Not Done	This patient was assigned to nurse with initials EF, this person did not write any score neither enter initials or completed documentation for NIGHT 25 under DRAIZE score.	No
101006	PureWick	12JAN2025	Subject Missed Follow-Up Visit	Nurse missed visit	No
	PureWick	13JAN2025	Clinical Assessment Not Done	missed pre weight documentation	No
101014	Hollister	19FEB2025	Clinical Assessment Not Done	Patient did not wear device	No
	Hollister	21FEB2025	Clinical Assessment Not Done	Nurse saw patient but Patient did not wear device	No
	Hollister	17MAR2025	Clinical Assessment Not Done	Visit was done but Patient did not wear device	No
	Hollister	15MAR2025	Clinical Assessment Not Done	Visit was done but Patient did not wear device	No
102001	PureWick	02NOV2024	Clinical Assessment Out of Window	Date of assessment entered day after baseline. Promis questionnaire Baseline dated 02/Nov/2024 day after baseline took place.	No
102006	PureWick	19NOV2024	Clinical Assessment Not Done	End of treatment questionnaire not administered.	No

Listing 16.1.5
Protocol Deviations
ITT

Subject ID	Planned Treatment	Date of Deviation	Nature of Deviation	Additional Details	Major Protocol Deviation
102011	Hollister	02JAN2025	Clinical Assessment Not Done	End of treatment questionnaire not administered.	No
102014	PureWick	27JAN2025	Clinical Assessment Not Done	subject did not wear device for night 1 due to miscommunication; she thought the nurse was supposed to come back to her home to place the device.	No
102020	PureWick	06MAR2025	Clinical Assessment Not Done	end of treatment questionnaires were not administered	No
	PureWick	11FEB2025	Other	on night one patient threw away bed pad, no post weight was obtained	No
103001	PureWick	12MAY2025	Subject Missed Follow-Up Visit	Participant was not available.	No
103002	Hollister	04MAY2025	Clinical Assessment Not Done	Subject did not use Bed Pad 1	No
	Hollister	04MAY2025	Clinical Assessment Not Done	PER INSTRUCTIONS OF CRA: 4 OUT OF 5 END OF STUDY QUESTIONNAIRES WAS NOT ADMINISTERED.	No
103003	PureWick	25MAY2025	Subject Missed Follow-Up Visit	Memorial Day - Nurse day-off.	No
105004	PureWick	21FEB2025	Subject Missed Follow-Up Visit	The subject missed using the device as she is not home. But the nurse restarted the in-home Clinical Assessment out of window from 22Feb2025 onwards	No
105006	PureWick	22FEB2025	Clinical Assessment Out of Window	The subject missed using the device as she is not home. But the nurse restarted the in-home Clinical Assessment out of window from 23Feb2025 onwards	No

Listing 16.1.5
Protocol Deviations
ITT

Subject ID	Planned Treatment	Date of Deviation	Nature of Deviation	Additional Details	Major Protocol Deviation
	PureWick	21FEB2025	Clinical Assessment Out of Window	The subject missed using the device as she is not home. But the nurse restarted the in-home Clinical Assessment out of window from 23Feb2025 onwards	No
105011	PureWick	27FEB2025	Clinical Assessment Not Done	The patient was not available for the assessment.	No
	PureWick	28FEB2025	Clinical Assessment Not Done	The patient was not available for the assessment.	No
105014	Hollister	13FEB2025	Other	There is not enough quantity of urine to transfer and weight	No
	Hollister	07MAR2025	Other	There is not enough quantity of urine to transfer and weight	No
	Hollister	06MAR2025	Other	There is not enough quantity of urine to transfer and weight	No
105015	PureWick	09FEB2025	Other	The Participant stated that she washed the underwear mesh and Purewick flex. so, the post weight for these were not entered in participant placement log.	No
105016	PureWick	19FEB2025	Clinical Assessment Not Done	Patient wants to take break from device use .	No
107004	Hollister	20FEB2025	Clinical Assessment Not Done	Draize scale not done with Night 2 , Nurse verbally inquired the subject about symptoms .	No
	Hollister	18MAR2025	Clinical Assessment Not Done	Draize score not done after Night 28, Nurse verbally inquired about symptoms .	No
	Hollister	17MAR2025	Clinical Assessment Not Done	Draize scale not done after Night 27, Subject was asked verbally about symptoms.	No
	Hollister	16MAR2025	Clinical Assessment Not Done	Draize score not done after Night 26 , Nurse verbally inquired about symptoms .	No

Listing 16.1.5
Protocol Deviations
ITT

Subject ID	Planned Treatment	Date of Deviation	Nature of Deviation	Additional Details	Major Protocol Deviation
	Hollister	15MAR2025	Clinical Assessment Not Done	Draize score not done after Night 25, Nurse verbally inquired about symptoms .	No
	Hollister	23FEB2025	Clinical Assessment Not Done	Draize scale not completed after Night 5 , Subject was inquired verbally about symptoms.	No
	Hollister	22FEB2025	Clinical Assessment Not Done	Draize scale not done after Night 4 , Nurse verbally inquired from subject about symptoms .	No
	Hollister	21FEB2025	Clinical Assessment Not Done	Draize scale not done with Night 3 , Nurse verbally inquired the subject about symptoms .	No
107005	PureWick	09MAR2025	Clinical Assessment Not Done	Draize skin score was missed after Night 28 , It was done at baseline and then the nurse inquired about the skin symptoms daily .	No
	PureWick	08MAR2025	Clinical Assessment Not Done	Draize skin score was missed after Night 27 , It was done at baseline and then the nurse inquired about the skin symptoms daily .	No
	PureWick	07MAR2025	Clinical Assessment Not Done	Draize score not performed after Night 26, Subject was inquired about symptoms verbally by the nurse .	No
	PureWick	06MAR2025	Clinical Assessment Not Done	Draize scale was missed after night 25. Performed at baseline and was followed by daily inquiries from subjects about symptoms if any.	No
	PureWick	14FEB2025	Clinical Assessment Not Done	Draize skin score was missed after Night 5 , It was done at baseline and then the nurse inquired about the skin symptoms daily .Bed pad 1 post weight not collected.	No

Listing 16.1.5
Protocol Deviations
ITT

Subject ID	Planned Treatment	Date of Deviation	Nature of Deviation	Additional Details	Major Protocol Deviation
	PureWick	13FEB2025	Clinical Assessment Not Done	Draize skin score was missed after Night 4 , It was done at baseline and then the nurse inquired about the skin symptoms daily .	No
	PureWick	12FEB2025	Clinical Assessment Not Done	Draize skin score was missed after Night 3 , It was done at baseline and then the nurse inquired about the skin symptoms daily .Bed pad 1 post use weight not collected.	No
	PureWick	11FEB2025	Clinical Assessment Not Done	Draize score was not done after Night 2 .Performed at baseline and then nurse inquiring daily about subject symptoms.	No
107007	PureWick	18MAR2025	Subject Missed Follow-Up Visit	Subject missed night 28 as she traveled.	No
	PureWick	17MAR2025	Clinical Assessment Not Done	Draize score not done after night 27 .Nurse inquired about symptoms from the subject.	No
	PureWick	16MAR2025	Clinical Assessment Not Done	Draize score not done after night 26,Nurse inquired about symptoms from the subject.	No
	PureWick	15MAR2025	Clinical Assessment Not Done	Draize score not done after night 25, .Nurse inquired about symptoms from the subject.	No
	PureWick	23FEB2025	Clinical Assessment Not Done	Draize score not performed after Night 5 , Only subject was inquired about the symptoms by Nurse.	No
	PureWick	22FEB2025	Clinical Assessment Not Done	Draize scale not done on nights 4 . Subject inquiries about symptoms	No
107010	Hollister	02MAR2025	Clinical Assessment Not Done	Nurse did not complete Draize score for , Night 5, Verbally inquired about the symptoms	No

Listing 16.1.5
Protocol Deviations
ITT

Subject ID	Planned Treatment	Date of Deviation	Nature of Deviation	Additional Details	Major Protocol Deviation
	Hollister	01MAR2025	Clinical Assessment Not Done	Draize score not completed for Night 4,Done at baseline and then Subject inquired about her symptoms daily by Nurse .	No
	Hollister	28FEB2025	Clinical Assessment Not Done	Nurse did not complete Draize scale for Night 3 , Verbally inquired about symptoms.	No
	Hollister	27FEB2025	Clinical Assessment Not Done	Draize score missed after night 2. Subject asked daily about symptoms by the nurse.	No
107016	PureWick	27FEB2025	Other	The subject mistakingly threw away the tubing and was unable to use the device. Supervisor is notified	No
107017	Hollister	02MAR2025	Other	Subject did not use the device due to adhesive not coming off and mild redness of the area.	No
107018	PureWick	10MAR2025	Clinical Assessment Not Done	Draize score not done for Night 4 , Nurse inquired verbally about symptoms daily .	No
	PureWick	09MAR2025	Clinical Assessment Not Done	Draize scale for Night 3 not done , subject was verbally asked about symptoms by the Nurse .	No
	PureWick	08MAR2025	Clinical Assessment Not Done	Draize score missed after Night 2 , Nurse verbally inquired about symptoms .	No
	PureWick	11MAR2025	Clinical Assessment Not Done	Draize score not done at Night 5 , nurse inquired the subject verbally about symptoms .	No
	PureWick	08MAR2025	Other	Subject did not use the device correctly as tubing came off and urine spilled, as per source note ,subject not able to put it together, Subject is old and was retrained by nurse.	No
	PureWick	07MAR2025	Other	Subject missed night 1 device placement.	No

Listing 16.1.5
Protocol Deviations
ITT

Subject ID	Planned Treatment	Date of Deviation	Nature of Deviation	Additional Details	Major Protocol Deviation
	PureWick	30MAR2025	Other	Subject misplaced the cylinder and was not able to use the device.	No
108001	Hollister	26FEB2025	Clinical Assessment Not Done	End of treatment questionaries was not completed per protocol.	No
	Hollister	25FEB2025	Clinical Assessment Not Done	Nurse did not measure the the Bed pad 1 pre-use weight and Bed pad 1 post -use weight	No
108002	PureWick	02MAR2025	Clinical Assessment Not Done	Nurse did not measure the Purewick Flex pre-use weight and post-use weight	No
	PureWick	01MAR2025	Clinical Assessment Not Done	Nurse did not measure the Purewick Flex pre-use weight and post-use weight	No
	PureWick	28FEB2025	Clinical Assessment Not Done	Nurse did not measure the Purewick Flex pre-use weight and post-use weight	No
	PureWick	27FEB2025	Clinical Assessment Not Done	Nurse did not measure the Purewick Flex pre-use weight and post-use weight	No
	PureWick	26FEB2025	Clinical Assessment Not Done	Nurse did not measure the Purewick Flex pre-use weight and post-use weight	No
108003	PureWick	28MAR2025	Subject Missed Follow-Up Visit	Subject did not wear the device on night 25.	No
	PureWick	27MAR2025	Subject Missed Follow-Up Visit	Subject did not wear the device on night 24	No
	PureWick	08MAR2025	Clinical Assessment Not Done	Nurse did not measure the Purewick Flex pre-use weight and post-use weight	No
	PureWick	07MAR2025	Clinical Assessment Not Done	Nurse did not measure the Purewick Flex pre-use weight and post-use weight	No
	PureWick	06MAR2025	Clinical Assessment Not Done	Nurse did not measure the Purewick Flex pre-use weight and post-use weight	No
	PureWick	05MAR2025	Clinical Assessment Not Done	Nurse did not measure the Purewick Flex pre-use weight and post-use weight	No

Listing 16.1.5
Protocol Deviations
ITT

Subject ID	Planned Treatment	Date of Deviation	Nature of Deviation	Additional Details	Major Protocol Deviation
	PureWick	04MAR2025	Clinical Assessment Not Done	Nurse did not measure the Purewick Flex pre-use weight and post-use weight	No
108004	PureWick	02MAR2025	Clinical Assessment Not Done	Nurse missed the assessmnet of Purewick Flex pre weight and Bad pad 1 post weight	No
	PureWick	01MAR2025	Clinical Assessment Not Done	Nurse missed the assessmnet of Purewick Flex pre weight and Bad pad 1 post weight	No
	PureWick	28FEB2025	Clinical Assessment Not Done	Nurse missed the assessment of Purewick Flex pre weight and Bed pad 1 post weight	No
	PureWick	27FEB2025	Clinical Assessment Not Done	Nurse missed the assessmnet of Purewick Flex pre weight and Bad pad 1 post weight	No
	PureWick	26FEB2025	Clinical Assessment Not Done	Nurse missed the assessment of Purewick Flex pre weight and Bad pad 1 post weight	No
108005	PureWick	04MAR2025	Clinical Assessment Not Done	Nurse missed the PureWick Flex pre weight and post weight assessment	No
	PureWick	03MAR2025	Clinical Assessment Not Done	Nurse missed the PureWick Flex pre weight and post weight assessment	No
	PureWick	02MAR2025	Clinical Assessment Not Done	Nurse missed the PureWick Flex pre weight and post weight assessment	No
	PureWick	01MAR2025	Clinical Assessment Not Done	Nurse missed the PureWick Flex pre weight and post weight assessment	No
	PureWick	28FEB2025	Clinical Assessment Not Done	Nurse missed the PureWick Flex pre weight and post weight assessment	No
111001	PureWick	20APR2025	Clinical Assessment Not Done	Draize conducted OOW due to patient being hospitalized on the day assessment was due. Eventually site went to patient in hospital and completed assessment on 26Apr2025.	No

Listing 16.1.5
Protocol Deviations
ITT

Subject ID	Planned Treatment	Date of Deviation	Nature of Deviation	Additional Details	Major Protocol Deviation
	PureWick	26APR2025	Clinical Assessment Out of Window	End of treatment questionnaire collected out of window due to pt being hospitalized at date of initial visit.	No
111003	Hollister	09MAY2025	Clinical Assessment Not Done	Post-weight for collection cannister was not obtained as there was no urine successfully collected.	No
	Hollister	07MAY2025	Clinical Assessment Not Done	Patient did not wear device overnight.	No
	Hollister	06MAY2025	Clinical Assessment Not Done	Patient did not wear device overnight.	No
	Hollister	16APR2025	Clinical Assessment Not Done	Patient did not wear device overnight.	No
114001	PureWick	17MAY2025	Clinical Assessment Not Done	N24 pre-weight not collected.	No
	PureWick	24APR2025	Clinical Assessment Not Done	N1 pre-weight not collected	No
	PureWick	24APR2025	Clinical Assessment Not Done	Baseline Draize not done.	No
	PureWick	25APR2025	Clinical Assessment Out of Window	Baseline Promis & N-Qol done after 1st night of use.	No
114002	Hollister	22APR2025	Clinical Assessment Not Done	Baseline Draize not done.	No
	Hollister	23APR2025	Clinical Assessment Out of Window	Baseline N-Qol & PROMIS done after baseline.	No
114003	Hollister	09MAY2025	Subject Missed Follow-Up Visit	Subject was sick & didn't wear device.	No
	Hollister	08MAY2025	Subject Missed Follow-Up Visit	Pt was out of town with husband. Didn't use device.	No

Listing 16.1.5
Protocol Deviations
ITT

Subject ID	Planned Treatment	Date of Deviation	Nature of Deviation	Additional Details	Major Protocol Deviation
	Hollister	07MAY2025	Subject Missed Follow-Up Visit	Subject was out of town with husband. Did not use device.	No
	Hollister	28MAY2025	Clinical Assessment Not Done	Night 24 Pre-weights not done.	No
	Hollister	05MAY2025	Clinical Assessment Not Done	N1 pre-weights not done.	No
	Hollister	05MAY2025	Clinical Assessment Not Done	Baseline Draize not done.	No
	Hollister	06MAY2025	Clinical Assessment Out of Window	Baseline, N-Qol & PROMIS done after baseline.	No
114004	PureWick	27MAY2025	Clinical Assessment Not Done	N24 pre-weights not done	No
	PureWick	04MAY2025	Clinical Assessment Not Done	N1 pre-weights not done	No
	PureWick	04MAY2025	Clinical Assessment Not Done	Baseline Draize not done.	No
	PureWick	05MAY2025	Clinical Assessment Out of Window	Baseline N-Qol & PROMIS done 5-5-25	No
114005	PureWick	29MAY2025	Clinical Assessment Not Done	N24 pre-weights not done	No
	PureWick	06MAY2025	Clinical Assessment Not Done	Baseline Draize not done	No
	PureWick	07MAY2025	Clinical Assessment Out of Window	Baseline N-Qol & PROMIS done after baseline	No
