# Supplementary figures and images for: Safety, Efficacy, and Patient-Reported Outcomes of the PureWick™ System Versus Comparator for Nocturnal Urinary Incontinence in the Home Setting: Results of a Randomized Trial
Source: J Clin Med. 2025 Dec 9;14(24):8699. doi: 10.3390/jcm14248699 (PMC12734071; doi:10.3390/jcm14248699)

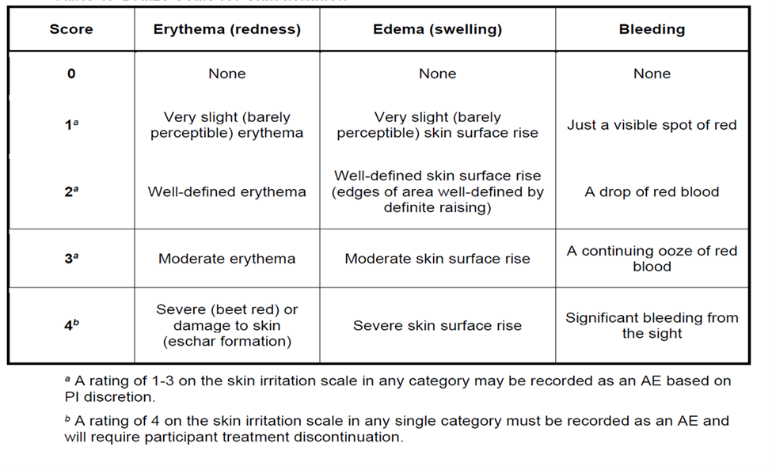

Supplement: Supplementary file 1 [file jcm-14-08699-s001.zip › S3_Draize scoring table.png]
